# Development and Refinement of HYlaMI, a Self-Hypnosis Training Program for Migraine Management: An ORBIT Phase Ia–Ib Acceptability Study

**DOI:** 10.3390/brainsci16101007

**Published:** 2026-09-23

**Authors:** Éloïse Cardinal, Léa Greban, Julie Delage, Nadia Godin, Valentyn Fournier, Jérémy Brunel, Mathieu Landry, Pierre Rainville, David Ogez

**Affiliations:** 1 Maisonneuve-Rosemont Hospital Research Center, Montreal, QC H1T 2M4, Canada; eloise.cardinal@umontreal.ca (É.C.); lea.greban03@gmail.com (L.G.); julie.delage.1@umontreal.ca (J.D.); ngodin.hmr@ssss.gouv.qc.ca (N.G.); valentyn.fournier@umontreal.ca (V.F.); jeremy.brunel@umontreal.ca (J.B.); 2 Department of Psychology, Faculty of Arts and Science, Université de Montréal, Montreal, QC H2V 2S9, Canada; 3 Faculty of Psychology, Université Libre de Bruxelles, 1050 Brussels, Belgium; 4 Department of Anesthesiology and Pain Medicine, Faculty of Medicine, Université de Montréal, Montreal, QC H3C 3J7, Canada; 5 Division of Hematology, Oncology and Transplantation, Maisonneuve-Rosemont Hospital, Montreal, QC H1T 2M4, Canada; 6 School of Psychology, Université Laval, Quebec City, QC G1V 0A6, Canada; mathieu.landry@psy.ulaval.ca; 7 Research Center of the Montreal University Institute of Geriatrics (CRIUGM), Montreal, QC H3W 1W5, Canada; pierre.rainville@umontreal.ca; 8 Department of Stomatology, Faculty of Dental Medicine, Université de Montréal, Montreal, QC H3A 1G1, Canada

**Keywords:** migraine, self-hypnosis, pain management, complementary intervention, psychophysiology

## Abstract

**Highlights:**

**What are the main findings?**

- After attending a presentation of the HYlaMI program and experiencing a single introductory hypnosis exercise, without receiving the program itself, participants reported limited prior knowledge of hypnosis, responded positively to the exercise, and expressed strong interest in continuing self-hypnosis and in taking part in the program.
- The single hypnosis exercise was followed by significant within-session improvements in self-reported relaxation, pain and anxiety (all *p* ≤ 0.004, r = 0.67–0.88), accompanied by non-linear changes in heart rate, breathing rate and nociception.

**What are the implications of the main findings?**

- Findings support the acceptability and relevance of structured, manualized self-hypnosis programs as a non-pharmacological complement to conventional migraine management and led to nine concrete refinements of the HYlaMI program.
- The results highlight the potential value of integrating hypnosis with standard medical care while addressing the unmet needs of people living with migraine, supporting further evaluation of hypnosis-based interventions.

**Abstract:**

**Background/Objectives:** Migraine is a prevalent and disabling neurological condition for which complementary non-pharmacological interventions remain underexplored. Hypnosis and self-hypnosis show promising potential for pain management; yet, structured, migraine-specific training programs remain limited. Following the ORBIT model, this Phase Ia–Ib study aimed to (1) define the HYlaMI program; (2) refine it through interviews with patients who received a detailed presentation of the program and experienced a single introductory hypnosis exercise drawn from it, without receiving the intervention itself, assessing perceived acceptability, benefits, limitations, and potential improvements; and (3) examine the within-session effects of that exercise on self-reported relaxation, anxiety, and pain, as well as on physiological activity. **Methods:** In this exploratory mixed-methods study, 17 adults living with migraines participated in a single-session study between September and December 2025. Participants were not exposed to the HYlaMI intervention at any point; they attended a single research session in which they received detailed information about the program and completed one hypnosis exercise drawn from it. Qualitative data were collected through semi-structured interviews, while quantitative measures included self-reported relaxation, anxiety and pain (rated verbally with the interviewer, who also delivered the exercise), and physiological activity (e.g., respiration and heart rate), assessed before, during and following the hypnosis exercise. **Results:** Four themes emerged: (1) Knowledge and impressions regarding hypnosis; (2) Relevance and acceptability of the program; (3) Expected and perceived effects of the program; and (4) Unanticipated elements. The fourth theme covers elements raised spontaneously by participants, notably migraine-related stigma and the ambivalence of relaxation. Participants showed strong enthusiasm for both hypnosis and the HYlaMI program. The hypnosis exercise was followed by significant improvements on numeric rating scales (0 to 10) for relaxation (M = 5.66 to 8.80), anxiety (3.28 to 0.88) and pain (2.81 to 1.31), all *p* ≤ 0.004 with large effect sizes (r = 0.67–0.88), alongside significant non-linear changes in heart rate, breathing rate and nociception. **Conclusions:** Findings support the acceptability of structured training programs based on hypnosis for migraine management and provide preliminary evidence of within-session benefits. They justify progression to ORBIT Phase II proof-of-concept and pilot studies testing the feasibility and preliminary efficacy of HYlaMI, and yield recommendations transferable to other non-pharmacological interventions for migraine.

## 1. Introduction

Migraine is one of the most common and disabling neurological conditions, affecting more than one billion individuals [1,2,3]. Although the sex difference in prevalence regarding this condition is expected to narrow, epidemiological projections estimate that prevalence will remain approximately twice as high in women [2]. Its global burden, which has been steadily increasing for more than 30 years, generates considerable direct and indirect repercussions at the individual and societal levels (e.g., reduced quality of life, mental health, and productivity), all of which are amplified by its strong comorbidity with other disabling conditions [1].

For patients, the management of migraines faces several major challenges: diagnostic errors, lack of access to specialized services, and inequitable access to appropriate treatments [1]. Furthermore, the exclusive use of medication presents significant limitations (e.g., side effects, contraindications, and risks of overuse) [4], while there is a lack of research on non-pharmacological approaches [5]. Altogether, these constraints underscore the need to develop and further integrate effective non-pharmacological interventions into treatment plans.

Migraine also differs from other chronic pain conditions in ways that limit the direct transfer of empirically supported psychological interventions. It is episodic and unpredictable, so that an intervention must be usable within minutes and without equipment, precisely when attending a supervised session is impossible [6,7,8]. Its sensory hyperexcitability (photophobia, phonophobia, osmophobia) makes the content of sensory suggestions non-neutral, since some correspond to recognised attack triggers [9]. Finally, relaxation, the usual proximal target of mind–body interventions for pain, is ambivalent in migraine, where attacks often arise during sleep or after a fall in stress [10]. Protocols validated in other pain conditions cannot therefore be assumed to transfer unchanged.

Medical hypnosis is an evidence-based complementary efficient treatment in numerous clinical contexts, including chronic pain [11,12], and appears promising for headaches and migraines [13,14,15,16]. Characterized by suggestion and dissociation techniques, which set it apart from other non-pharmacological interventions, hypnosis provides a non-invasive, low-cost, and widely accessible approach that requires little equipment and time [17,18,19].

Three recent clinical studies involving clinical hypnosis report improvements in migraine-related disability, pain-related psychological factors (e.g., catastrophizing), and characteristics of attacks (e.g., intensity, duration) [4,20,21]. Nevertheless, the body of evidence remains limited and methodologically heterogeneous. Recent reviews converge on a consistent set of shortcomings: small and generally unpowered samples, frequent absence of an active or credible control condition, wide variability in the number, duration and content of the hypnosis sessions delivered, incomplete reporting of the hypnotic protocols themselves, and inconsistent outcome selection [22]. Together, these limitations preclude replication, prevent meaningful pooling of results, and make it impossible to determine which components of a hypnosis intervention drive any observed benefit. Addressing these shortcomings requires structured programs that would specify what is delivered, to whom and in what order, and that are built through staged development frameworks rather than tested directly in efficacy trials [15,22,23]. Here, learning self-hypnosis (the autonomous use of hypnotic exercises) appears particularly well-suited to the unpredictable context of migraine attacks, affording patients autonomy in practice and the potential for more lasting effects [24,25].

A self-hypnosis training program for chronic pain (HYlaDO), developed in Montreal (Quebec, Canada), rests on a eight weekly one-hour group sessions via teleconsultation and the combination of hetero-hypnosis (hypnosis guided by a practitioner) and self-hypnosis practice at home using customized recordings [26]. A recent non-randomized pre-post study of this training program in the context of chronic pain reported multifactorial benefits, with significant short-term reductions in pain and anxiety and a significant short-term increase in relaxation [27]. Preliminary results further suggested improvements in quality of life. Similarly beneficial results were reported in a qualitative proof-of-concept study of the HYlaDO intervention [28]. Building on these promising results, we relied on HYlaDO’s core components for the development of a self-hypnosis training program in the context of migraine.

Monitoring physiological activity at this stage is motivated by two lines of evidence. Hypnotic responding is accompanied by a shift towards parasympathetic predominance, reflected in heart rate and breathing rate, and by cortical changes that are only partly attributable to the formal induction [29]. Interoception is also of interest in migraine, where interoceptive accuracy is preserved but sensibility is altered [30], and where the cortical processing of cardiac afferents is modulated by hypnotisability and by hypnosis [31]. Monitoring cardiac, respiratory and nociceptive indices during a first exercise thus documents whether it engages the systems through which hypnosis is thought to act.

HYlaMI (HYpnosis for Migraine, in French, *HYpnose pour la MIgraine*) is a novel self-hypnosis training program, designed to support migraine management. Importantly, the present study did not deliver the HYlaMI intervention. Participants attended a single research session in which they received a detailed presentation of the program and experienced one introductory hypnosis exercise drawn from it; no other component of the program was administered. The exercise was not an unrelated analogue: it is the *Pleasant Place* exercise of Session 1, delivered verbatim from the program script, and it carries the active ingredients shared by all seven exercises (induction, deepening, mind–body dissociation and post-hypnotic suggestions). It is thus the smallest testable component of HYlaMI. This study served three main objectives: (1) to define the HYlaMI program; (2) to refine it through participants individual interviews, collecting data on its perceived social acceptability, benefits, limitations, and potential improvements; and (3) to examine participants’ experience of a single-session hypnosis exercise drawn from the program, via self-reported current relaxation, anxiety, and pain, and to assess concurrent physiological changes, in order to evaluate the feasibility and relevance of physiological monitoring during future intervention sessions. This study enables progression to the next phases of intervention development, before the assessment of efficacy in subsequent pilot and efficacy trials [32].

## 2. Materials and Methods

### 2.1. The ORBIT Model

To adapt an existing program to a new clinical context, it is recommended to follow formalized development and evaluation models. To this end, the ORBIT model proposes four phases of development and particularly emphasizes the importance of the first two: phase I, comprising program definition (Ia) and refinement (Ib), and phase II, newly divided into three sub-phases in the latest version of the model, consisting of a proof of concept (IIa) evaluating the program’s capacity to produce clinically significant changes, a pilot and feasibility study (IIb) evaluating feasibility and preliminary effects in a small sample, and a phase II efficacy study (IIc) (see Figure 1) [32,33]. These phases precede the randomized controlled trials of phases III and IV. The present study addresses both stages of phase I of development: Ia (definition) and Ib (refinement).

### 2.2. Phase ORBIT Ia: Definition of the HYlaMI Program 

To answer our first objective (1), we developed the HYlaMI program based on the self-hypnosis program for chronic pain (HYlaDO), retaining the same structure of eight weekly one-hour teleconsultation sessions organized around seven complementary exercises targeting the physical and emotional dimensions of pain: four aimed at developing internal resources (e.g., pain acceptance, stress and anxiety management) and three aimed at modifying pain perception. We tailored the fourth session more specifically to migraine by replacing its original hypnosis exercise, the *Control Room*, with the *Thermal Helmet*, a pain-perception exercise incorporating suggestions of thermal regulation of pain, previously used in studies on migraine and hypnosis [22].

Each exercise follows a structured framework (induction, deepening, hypnotic work, post-hypnotic suggestions, and return to waking state) and was pre-recorded in a studio to allow participants to practice self-hypnosis at home. The first seven sessions each include one hypnosis exercise, and the 8th session is one of conclusion. The 1st session introduces the program with a psychoeducation presentation on medical hypnosis, its use in pain and migraine management and a short presentation of the program. It concludes with a first hypnosis exercise, the *Pleasant Place*, inviting participants to revisit a location of their choice through their five senses to generate relaxation and a sense of calmness. The 2nd session, the *Backpack*, aims to work on the management of anxiety, emotions, and stress. It incorporates a metaphor of a bag in which participants must sort through rocks symbolizing negative emotions and daily discomforts, and feathers symbolizing available resources and the possibilities of feeling better and being happy. The 3rd session, *Doing Nothing*, works on acceptance and the reduction of pain catastrophizing through suggestions of letting go and meditation in a pleasant place. The 4th exercise, the *Thermal Helmet*, uses visualization to modify the perception and intensity of pain in the cephalic region. It is suggested that the pain takes the form of an image that the patient can modify in terms of its shape or intensity. The 5th session, the *Magic Hand*, consists of creating through suggestions a magic hand capable of relieving discomfort in any part of the body where it is placed. In the 6th session, the *Rossi Method*, participants are invited to symbolize their positive resources in one hand and their discomfort in the other, and then, by bringing both hands together, to relieve their pain. The 7th and final exercise, *Reconnecting with a Resource*, guides the patient to recall past experiences of managing pain and difficult emotions, to enhance awareness of these resources and build a personal repertoire of self-management tools that can be drawn upon whenever needed. Finally, in the 8th session, *Closing*, the exercises are revisited, participants’ experiences are discussed, and self-hypnosis is prescribed.

Following the International Headache Society’s guidelines [34], the primary therapeutic target of HYlaMI was chosen to be migraine-attributed burden (including migraine-related disability, its impacts, and its impacts on quality of life) [35], measured with the HIT-6 and the MIDAS [36,37]. Additionally, several secondary targets were planned: anxious-depressive symptoms (HADS), current relaxation, anxiety and pain (using numeric rating scale; NRS), migraine frequency, intensity, and duration, and medication use (using participant diary) [38].

### 2.3. Phase ORBIT Ib: Refinement of the HYlaMI Program and Preliminary Effects

This single-session qualitative study was conducted individually with participants living with migraine, during which they received detailed information about the HYlaMI program and were then exposed to one hypnosis exercise drawn from it. Participants did not take part in the HYlaMI program itself, and no component of the program other than this single exercise was delivered to them. It aimed to answer our second and third objectives: (2) to evaluate the social acceptability, advantages, limitations, and areas for improvement of the program and a hypnosis exercise, through individual interviews with participants; and (3) to examine the effects of a hypnosis exercise, drawn from HYlaMI, on current relaxation, anxiety, pain, and certain physiological markers (heart rate, breathing, level of consciousness, and nociception). The qualitative method was appropriate in this context, as it allows for the emergence of a critical perspective, enables in-depth exploration of the phenomenon, and facilitates rich analysis of data collected from a small sample [39,40].

**Participants.** Seventeen individuals living with migraines were included in this study. They had a formal diagnosis of migraine for more than one year (issued by a physician), spoke French, and did not consume recreational drugs or alcohol on the day of the study. Participants reported all medications currently taken, including migraine-specific treatments, other analgesics and non-analgesic medications, and were asked to indicate whether any had been taken on the day of the session. Participants who took acute migraine or analgesic medication during the study session itself, before the hypnosis exercise, were retained in the qualitative analyses but excluded from the quantitative analyses. Exclusion criteria included having issues that interfere with comprehension (e.g., deafness), severe unstabilized neurological or psychiatric disorders that impair communication, or participating in another psychological intervention at the time of the study.

**Materials and Procedures.** Participants were recruited through the Facebook page and newsletter of Migraine Québec, a non-profit organization to help people with migraine. Interested individuals contacted the research coordinator by email, confirmed their eligibility, and were offered a time slot for their hospital visit. The first 17 participants confirming their participation were included. No power calculation was performed, none being applicable at this stage: the deliverable of ORBIT Phase Ib is a refined intervention, and the governing criterion is informational rather than statistical [32,33]. Seventeen participants lies within the range recommended for a thematic analysis of a focused topic in a homogeneous population. Recruitment through a patient organisation was deliberate, this phase requiring participants able to critique the program; the resulting self-selection is addressed in the Limitations.

A few days before their hospital visit, selected participants received by email a reminder of their visit date containing all logistical information, a sociodemographic and clinical questionnaire to complete and return to the research coordinator, as well as the consent form, so that they could familiarize themselves with it. On the day of the visit, the research coordinator answered their questions prior to the signing of the free and informed consent form. The hospital visit, lasting a total of 90 min, was divided into 4 steps: (1) the signing of participants’ consent; (2) a lecture with visual support, planned to be presented during the program’s first session, lasting approximately twenty minutes and covering medical hypnosis, neuroscience data and clinical studies, its application in migraine management, and the HYlaMI program; (3) the installation of monitoring equipment; and (4) the hypnosis exercise (see Figure 2).

**Description of measurements**. At three key moments during the session, participants were asked questions about their perception of medical hypnosis (Q1, before the presentation), the HYlaMI program (Q2, after the presentation), and their experience of hypnosis (Q3, after the exercise). These individual interviews of 15 to 30 min, conducted by ÉC, aimed to identify desirable and undesirable effects as well as areas for improvement of the program. The interview guide was adapted from the Semi-Structured Interview for Social Validity Assessment, consisting of funnel questions evaluating the three components of social validation: social importance of goals, acceptability of procedures, and importance of effects [41]. This method has proven useful in other studies seeking to evaluate the social validity of new interventions [42,43]. The interview guide covered the following five general themes: impressions of hypnosis, relevance and acceptability of the program, experience of hypnosis, expected and perceived effects, recommendations; each containing sub-questions (English translation in Supplementary Material File SM1). The interviews were audio-recorded, stripped of any identifying information, and transcribed via WhisperAI [44] prior to deletion of the recordings. The verbatim transcripts were then verified by a member of the research team to detect any artifact (JD).

**Hypnosis Exercise and Physiological Monitoring**. During the installation of the monitoring equipment (by research assistants LG or AL, in acknowledgments), sensors were placed on participants to assess physiological activity. Using the Dräger Infinity C700 (Drägerwerk AG & Co. KGaA, Lübeck, Germany), respiratory rate and heart rate were monitored [45]. Three disposable Kendall electrodes with Conductive adhesive hydrogel (530 Foam Electrodes, REF 31013926) were placed on the participant’s left clavicle, right clavicle, and left thoracic flank. Using a PMD-200™ device from Medasense Ltd. (Ramat Gan, Israel) and an electrode placed using a painless clip on the middle finger, the NOL^®^ Index was used to monitor the participant’s nociception level [46]. Finally, a Bispectral Index™ (BIS™) monitoring system and a BIS™ extension sensor comprising 4 electrodes, applied to the forehead, were used to monitor the electrical activity (EEG) of participants’ frontal cortex and provide a direct measure of the patient’s level of consciousness [47].

The NOL^®^ Index is a unitless composite score from 0 to 100, computed by a non-linear model combining photoplethysmography, galvanic skin response, skin temperature and accelerometry acquired at the fingertip; validated in the operating room against graded noxious stimulation, it indexes the autonomic response to nociceptive input rather than the subjective experience of pain. The BIS™ is a processed-EEG index from 0 (isoelectric EEG) to 100 (fully awake), derived from the frontal EEG and validated as a measure of depth of hypnosis under general anaesthesia. Heart rate, breathing rate and the NOL were expected to decrease with the shift towards parasympathetic predominance that accompanies hypnotic relaxation, and the BIS was included to index the associated cortical changes. Both indices were, however, developed under general anaesthesia, where sedation is far deeper; their behaviour in awake participants under hypnosis is not established, and testing their usability was itself an objective of this monitoring.

Then, participants experienced the *Pleasant Place* hypnosis exercise (the exercise scheduled for Session 1 of HYlaMI; Session 1 itself was not delivered) for approximately 30 min, guided by ÉC, a doctoral candidate in neuropsychology who has been trained in medical hypnosis for 4 years (English translation in Supplementary Material File SM2). In the exercise, participants were asked to explore a place or a memory of their choosing using the VAKOG technique (visual, auditory, kinesthetic, olfactory, gustatory). It incorporated mind-body dissociation suggestions, allowing the mind to wander while the body regenerated, and to let go of past or present tensions that were no longer useful to the person. Before (T1) and after the exercise (T2), participants completed three numeric rating scales (NRS) from 0 to 10 assessing their state of relaxation, anxiety, and level of pain (induced by migraine and/or other present health conditions).

Physiological signals were recorded continuously from the beginning of the induction to the end of the return to the waking state. Because the exercise was delivered in a permissive, Ericksonian style and paced to each participant’s own responses, the boundaries between its components (induction, deepening, hypnotic work, post-hypnotic suggestions, return to the waking state) were not identical across participants, and the recordings were therefore not epoched with respect to those components. For analysis, each participant’s recording was instead divided into five consecutive segments of equal duration (time 1 to time 5), and the mean of each physiological index was computed within each segment. These segments index the overall temporal evolution of each signal across the exercise and do not correspond to specific hypnotic phases or suggestions; this constraint is revisited in the Limitations. The full verbatim of the exercise, including the categories of suggestion used, is provided in Supplementary Material File SM2.

### 2.4. Analysis

Descriptive analyses were conducted on the sociodemographic data. For the qualitative data, a thematic analysis using continuous thematization [48] was conducted by two members of the team (ÉC, woman, 27 y.o., PhD candidate in Clinical neuropsychology; JD, woman, 46 y.o., PhD student in Psychology) to ensure inter-rater agreement [49,50]. The team already has considerable experience in the use of such qualitative methodologies [42,43]. The transcript analysis followed these steps: (1) importation of the transcripts into the qualitative analysis software NVivo 15 [51]; (2) creation of the preliminary thematic tree and description of anticipated codes, based on the interview questions; (3) sequential coding of transcripts by both analysts individually (JD, then ÉC); (4) a team meeting to evaluate inter-rater agreement, adjustment of the thematic tree and code descriptions based on the emergence of new themes (ÉC, JD); (5) re-coding of transcripts using the new tree; (6) a team meeting to present the final results (ÉC, JD, DO); (7) creation of a summary table of preliminary results incorporating notable quotes. The final thematic structure, with the definition of each theme and sub-theme, is provided in Supplementary Material File SM3. Alphanumeric numbers were assigned to participants (P1 to P17) to reference them in the narrative of qualitative results, and the verbatim passages presented were minimally modified while preserving their meaning and removing superfluous content (use of […]). The Confluence software was used for the schematic illustration of the thematic tree [52].

**Effects of the Hypnosis Exercise.** Quantitative analyses, derived from the NRS (relaxation, anxiety and pain) and physiological measurements, were also conducted. To analyze the perception of current relaxation, anxiety and pain measured using NRS during the hypnosis exercise, non-parametric comparisons between scores before (T1) and after (T2) the hypnosis exercise were conducted using the paired Wilcoxon test (*p* = 0.05) [53] in R Studio 2026.08.1+195 [53].

To assess the effect of the hypnosis exercise procedure on the physiological components, we assessed the changes in the heart rate by minute (HR), breathing rate (BR), nociceptive level (NOL) and level of consciousness (BIS) throughout the hypnosis work and deepening. The hypnosis procedure was delivered by ÉC and aimed at being adapted to the participant temporality and self-integration of the exercises. The steps of the procedure were not epoched. Nevertheless, each hypnotic session was divided into 5-time measurements by participant (time 1 to time 5), enabling to conduct a longitudinal analysis assessing different evolutive curvatures (linear, quadratic, cubic) of these components throughout the procedure. The mean of each segment was taken as the dependent variable. Analyses were conducted through linear mixed models with the package lme4 [54] in Rstudio and using *p* = 0.05 as the significant criterion. Three polynomial slopes were considered as fixed effects: (1) linear, assuming a constant decrease or increase of the components with hypnosis deepening, (2) quadratic, assuming a parabolic decrease or increase of the components, and (3) cubic, assuming a S-shaped decrease or increase of the components. This is a standard polynomial growth-curve specification for repeated measures; the three terms are orthogonal, and each was tested separately for each variable [55]. A significant linear term indicates a monotonic change, a quadratic term one inflection, and a cubic term two inflections (an S-shaped trajectory). Since the segments were obtained by equal division rather than by alignment with protocol events, these terms describe the overall evolution of each signal and cannot be attributed to specific hypnotic phases or suggestions. Random intercept and slopes for the three polynomial slopes were considered as the level of participant. Correlations between random effects were allowed. Data from one participant were not considered for the breathing rate analysis due to extreme data points. Effect sizes were computed using the f^2^ statistic derived from the beta coefficient and the standard error of the fixed effects, following the procedure for linear mixed models proposed by Groß and Möller, in which f^2^ is obtained from the Wald statistic of each fixed effect (the ratio of the coefficient to its standard error) without requiring a coefficient of determination for the model, and interpreted as small (f^2^ ≈ 0.02), medium (f^2^ ≈ 0.15) or large (f^2^ ≈ 0.35) following conventional benchmarks [56].

## 3. Results

### 3.1. Description of the Sample

A total of 17 participants were recruited and completed the study. All were included in the qualitative analysis. Data from one participant were removed from the quantitative analysis due to her having taken migraine pain medication during the hospital session, prior to the hypnosis exercise (see Figure 3). Qualitative analyses therefore concern all 17 participants, whereas quantitative analyses concern the remaining 16. The detailed description of the sample is provided in Table 1. Fifteen participants were women and two were men. Mean age was 55.88 years (SD = 12.11; median = 51.00; range 32–71). Most were Caucasian (*n* = 16), in a committed relationship (*n* = 13, of whom 8 were married), and had attained a university level of education (*n* = 10). Most had no children (*n* = 5) or two children (*n* = 7) and were either retired (*n* = 5) or on work disability (*n* = 7). Most suffered from chronic migraines (headache occurring on 15 or more days/month for more than 3 months, which, on at least 8 days/month, has the features of migraine headache) [57], with an average of 13.75 migraine days per 30 days (SD = 10.26; median = 12.00; range 1–30), for which they all took medication. They had been living with migraine for an average of 22.35 years (SD = 16.73; median = 20.00; range 2–60), and most reported a family history of migraine (*n* = 12). On average, they rated their usual daily pain level, all causes combined, at 6.56/10 (SD = 1.75; median = 6.50; range 2.50–10.00) and their usual daily anxiety level at 5.28/10 (SD = 2.29; median = 5.75; range 1.00–8.00; *n* = 16). Finally, eight participants reported a comorbid psychological disorder (anxiety, *n* = 4; depression, *n* = 1; post-traumatic stress disorder, *n* = 1; complex post-traumatic stress disorder, *n* = 1).

### 3.2. Qualitative Analysis of the Interviews

From the analysis of the interview transcripts, four main themes emerged: (1) Knowledge and impressions regarding hypnosis; (2) Relevance and acceptability of the program; (3) Expected and perceived effects of the program; and (4) Unanticipated elements (see Figure 4). The four themes are summarized in Table 2, and selected verbatim quotations illustrating each theme and sub-theme are provided in Supplementary Material Table SM5.

#### 3.2.1. Theme 1: Knowledge and Impressions Regarding Hypnosis

This theme encompasses participants’ perception of hypnosis: their prior knowledge and experiences, their impressions of this method following the presentation of the program and then following the experience of a first hypnosis exercise.

***Prior knowledge and experience with hypnosis*** (17/17). Most participants reported little knowledge of hypnosis beyond media portrayals and stage shows (e.g., P17), and some required a scientific framework before trusting the method (e.g., P14). Others nonetheless distinguished medical from stage hypnosis and identified its therapeutic uses for pain, anxiety, stress and sleep. Prior exposure came from online videos and podcasts, television, psychological consultations, or hospital continuing education (e.g., P3); those with previous experience described it as positive and deeply relaxing.

***Impressions/attitude toward hypnosis after the program presentation*** (13/17). The lecture, and particularly its description of scientific studies, lent legitimacy to hypnosis and reinforced participants’ positive perception of it (e.g., P14). One participant remained cautious for lack of direct experience, while open to trying the practice (P16).

***Experience/views on hypnosis during/after the hypnosis exercise***

***Views on hypnosis after the exercise*** (12/17). Most participants viewed hypnosis positively after the exercise, describing it as safe, non-invasive (e.g., P7) and usable as a personal tool; one (P15) noted its similarity to meditation.

***Change in views on hypnosis following the experience*** (14/17). Most already held a favourable opinion. The exercise mainly confirmed the expectations formed after the lecture, while revealing hypnosis to be simpler than anticipated and an intrinsic human capacity (e.g., P14). One participant (P6) who had doubts reported a positive shift.

***Sensations experienced during/after the exercise*** (16/17). Participants described marked bodily changes: heaviness, release of tension, immobility and an overall slowing down, several likening the state to several hours of sleep (e.g., P12). One participant who meditates daily felt more relaxed than during her usual practice (P17). A few reported reduced discomfort and pain; one (P17), in an acute migraine episode during the session, reported feeling no pain at the deepest point of the hypnotic state. Mental changes were equally salient: inner calm, a cessation of the incessant flow of thoughts allowing a refocusing on oneself and a detachment from daily life (e.g., P3), a sense of safety and control throughout, and rich multisensory imagery—vivid colours, smells, the sensation of wind—described by one participant as a true “mental journey” (P1, P8). Only one participant (P14) reported increased discomfort, due to resistance to certain suggestions (see Section 3.2.2).

***Adherence to the method following the exercise*** (6/17). All participants who commented expressed interest in repeating the experience and in learning self-hypnosis as an autonomous tool for managing their migraines and associated symptoms, including anxiety (e.g., P6).

#### 3.2.2. Theme 2: Relevance and Acceptability of the Program

This theme explores participants’ perception of the relevance and acceptability of the program for migraines, touching on their overall impression of the program, its content (e.g., exercise content), and its structural aspects (e.g., session duration). Participants also offered recommendations.

***Impressions of the program*** (17/17). Participants described the program as interesting, well-structured, relevant and comprehensive (e.g., P14). The lecture made the intervention more concrete and engaging. Some perceived it as a toolbox well adapted to migraine, with the potential to help them manage their emotions and pain autonomously (e.g., P8); one felt that “everything had been thought of” (P10). Others emphasized the hope it brought when they believed they had tried everything. All expressed a desire to take part.

***Number of sessions*** (14/17). Eight weekly sessions were considered appropriate, sufficient and adapted to participants’ reality (e.g., P16). One participant (P8) noted that this number promotes adherence, since at least one session is likely to coincide with an attack and thereby demonstrate the value of hypnosis directly. No participant wished for fewer than eight sessions, and some would welcome more (e.g., P17); one (P4) considered eight insufficient for chronic migraine and would prefer an intervention spread over four to five months across two seasons.

***Duration of sessions*** (13/17). One hour was seen as the ideal session duration, comparable to other medical appointments (e.g., P5): long enough to be effective without exhausting people living with chronic pain. Participants added that this amount of screen time is acceptable given the option to close one’s eyes and focus solely on the practitioner’s voice.

***Therapeutic targets of the program*** (13/17). Migraine-attributed burden was unanimously endorsed as the primary target: measurable, applicable to all migraine profiles, and able to capture disabling symptoms beyond pain (fatigue, sensory hypersensitivity, cognitive impairment, nausea), thereby reaching people whose migraine is not predominantly painful, such as vestibular migraine (e.g., P7). This inter-individual variability nonetheless led P14 to question the feasibility of a personalized measure of burden.

Psychological factors were also identified as essential targets, P4 judging them more appropriate than pain perception because of their potentially more lasting effects. Examples included anticipatory and performance anxiety, depression, guilt related to loss of functioning, and discouragement in the face of therapeutic failures (e.g., P5). Two participants would prioritize migraine frequency.

***Relevance of the timing of hypnosis or intervention delivery.*** Participants shared their views on the best times to conduct the intervention and practice self-hypnosis.

***Best time of day for delivering the intervention*** (15/17). No consensus emerged, delivery time depending on circadian rhythm, migraine profile, obligations and energy levels. Late morning (10 a.m.–12 p.m.) stood out as the best compromise, avoiding morning attacks while preserving concentration and energy (e.g., P12), followed by early afternoon (12–4 p.m.) and the evening (7–9 p.m.). Early morning (before 9–10 a.m.) and late afternoon (after 4–5 p.m.) were to be avoided. P9 suggested offering scheduling flexibility.

***Best timing for administering hypnosis*** (5/17). Two participants (P3, P6) saw hypnosis as useful at every phase of migraine. Three (P4, P5, P9) identified the prodromal phase as the preferred window, potentially reducing anticipatory anxiety and delaying or interrupting the attack. During an attack, hypnosis could divert attention and modify pain perception (P6, P9); in the postdromal phase, it could promote a return to calm, energy recovery and prevention of a subsequent attack (P3, P6).

***Choice of hypnosis exercises*** (16/17). Participants considered the range of exercises comprehensive, relevant and well adapted to the different facets of living with migraine. The *Magic Hand*, the *Pleasant Place*, the *Backpack* and *Doing Nothing* were particularly highlighted, as was the use of visual metaphors during sessions.

Participants also recommended therapeutic themes for future exercises. P3 suggested sessions focused on post-attack recovery, to re-energize the body and target physical hypersensitivities that exacerbate sensory irritability during an attack. Three participants (P2, P10, P15) noted that exercises should account for migraine affecting the neck and shoulders and not only the head, and recommended psychoeducation on energy management, in particular the importance of pacing oneself on good days (e.g., P10).

***Relevance of exercise content*** (8/17). Content was judged broadly appropriate, though several participants identified suggestions and metaphors that triggered internal defence mechanisms. P4 and P14 recommended avoiding references to parents as figures of comfort, which may reawaken family tensions, and any wording evoking typical migraine triggers (e.g., the word “perfume”), in favour of neutral imagery. P14 found the suggestion to forget past tensions too categorical and proposed welcoming pain rather than erasing it; she also reported that the countdown used during deepening induced anticipatory anxiety, suggesting a staircase-descent metaphor instead, and recommended imagery of water enveloping the person and dissolving pain and apprehension.

Two participants (P14, P17) reported anxiety in response to relaxation suggestions, because most of their attacks begin at night; P14 attributed a negative connotation to relaxation, feeling she must remain vigilant to wake in time to take her medication. Finally, P2 recommended encouraging participants to adapt their physical environment, and P3 and P4 stressed the diversity of individual needs (e.g., difficulty with imagery, difficulty holding a single mental image when anxious), arguing for adaptable exercises.

***Number of participants*** (15/17). A group of eight (ten maximum) was considered optimal, allowing everyone to share while preserving intimacy in a virtual format (e.g., P12), provided the framework limits exchanges during the hypnotic session itself. Two participants (P3, P12) valued the group for its diversity of perspectives and mutual support; one (P2) preferred six, while acknowledging the cost in accessibility.

***Telepractice delivery modality*** (17/17). Telepractice was seen as essential to accessibility, allowing people in rural areas or amid an attack to participate without travelling and limiting cancellations (e.g., P1). It also allows participants to adapt their own environment (lighting, position, comfort, noise) and reduces anticipatory stress (P13). One participant (P6) raised screen fatigue and the feeling of being watched, but the advantages were considered predominant. Three participants (P10, P15, P16) preferred in-person sessions, and P3 questioned whether the two modalities are equally effective.

***The ideal clinician*** (17/17). Vocal characteristics were decisive: a calm, soft, pleasant voice and slow delivery (e.g., P10). In-depth knowledge of migraine and adequate hypnosis training were also considered necessary, the practitioner’s profession mattering little. Valued personal qualities included attentiveness, benevolence, patience and comfort with pain. One participant (P15) would prefer a female practitioner, given the predominance of the condition among women.

***Suggestions for improvement*** (5/17). P15 proposed opportunities for exchange between participants, to build community and counter the isolation this population experiences, and recommended make-up sessions for those who miss a session because of an attack, each session ideally being offered twice, with audio recordings only as a last resort. P14 suggested grouping participants by similar pain or migraine profile, and making supplementary resources available for those wishing to deepen their knowledge of hypnosis. Two participants (P4, P14) proposed a post-intervention follow-up session to support continued practice and reduce the feeling of abandonment at the end of treatment.

***Other comments*** (7/17). A controlled sensory environment (dim lighting, silence, no strong odours or perfumes, comfortable temperature) was considered critical should in-person sessions be planned, both to promote relaxation and to avoid triggers. Three participants (P2, P3, P9) regarded the audio recordings as an essential component, enabling autonomous practice between sessions, serving as a catch-up plan after a missed session, and constituting a distinctive advantage over other available treatments.

#### 3.2.3. Theme 3: Expected and Perceived Effects of the Program

This theme covers two distinct sets of effects that participants were asked about separately: the effects they anticipated from the HYlaMI program based on the presentation they had attended, and the effects they reported experiencing during the single hypnosis exercise. Because participants had not received the program, all program-level effects reported below are anticipated rather than experienced.

***On anxiety-depressive symptoms and stress*** (16/17). The intervention was seen as an effective means of reducing stress and anxiety, both preventively and during an attack, by interrupting the anxiety spiral through refocusing and relaxation. During an attack, it would divert attention and lessen the psychological burden of pain, indirectly reducing its intensity and improving adaptive capacity (e.g., P12). Participants expected broader effects on mood, motivation and daily functioning, with fewer negative thoughts, less anticipation of attacks, and a greater sense of security and resilience.

***On relaxation*** (12/17). The intervention was seen as an effective tool for both physical and mental relaxation, providing sensations of calm, mental stability and bodily ease. Some participants identified this physical relaxation as particularly beneficial after an attack, to aid post-attack recovery (e.g., P4).

***On pain, its management, or perception*** (7/17). Participants anticipated a temporary reduction in pain, experienced as immediate relief (e.g., P11), but described hypnosis as a management tool rather than a cure: partial and temporary in its effects, yet functionally significant. They particularly expected an improved ability to tolerate and cope with pain day to day, a concrete benefit for a chronic and often refractory condition (e.g., P10).

***On medication use*** (14/17). Participants saw the intervention as a potential means of reducing medication use by offering a non-pharmacological alternative, a prospect particularly valued in a context of recurrent consumption. Hypnosis was nonetheless envisaged as a complement rather than a substitute, to be favoured when they wish to delay or avoid taking medication (e.g., P13).

***On migraine frequency, intensity, or duration*** (13/17). Several participants anticipated a reduction in migraine frequency and intensity, mainly through indirect effects such as reduced anxiety, increased relaxation and less anticipation of attacks, with a consequent decrease in medication use and a perception of greater treatment effectiveness when medication is used (e.g., P9). One participant (P13) remained reserved about any direct and measurable impact, emphasizing the need for empirical data on long-term effects.

***On daily functioning*** (16/17). Building on the other anticipated effects, participants expected improved daily functioning, making it easier to carry out everyday activities despite residual symptoms, and more broadly an improved quality of life—moving through each day with greater ease and less daily suffering (e.g., P7).

***Other expected or perceived effects*** (2/17). P14 believed regular self-hypnosis could strengthen the sense of inner security that is difficult to maintain in chronic pain, while P1 highlighted its potential for people who have experienced trauma, by allowing them to transform their narrative positively.

#### 3.2.4. Theme 4: Unanticipated Elements (9/17)

Nine participants raised elements not anticipated in the interview guide, in two areas. First, five participants (P3, P5, P13, P14, P15) reported a profound sense of being misunderstood, both by those around them and by the medical system. They described the trivialization of migraine, often reduced to a simple headache, and the discomfort that chronic pain elicits in loved ones, leaving them alone with their condition and reluctant to speak for fear of burdening others (e.g., P15). Several explicitly attributed this to the stigmatizing history of migraine, tied to the gendered nature of a predominantly female condition, which had hindered research and the legitimization of treatments. One participant (P15), and she alone, proposed renaming the condition to break free from this legacy.

Second, three participants (P1, P12, P15) expressed weariness with a medical approach focused almost exclusively on medication, whose effectiveness they often found disappointing. One advocated better dialogue between complementary approaches and neurologists, feeling these two worlds still operate in silos, and the intervention was perceived as a welcome response to a self-management need that conventional medicine does not fully address (e.g., P15). Three further participants (P9, P10, P12) stressed the importance of broader dissemination among the public, healthcare professionals and organizations such as Migraine Québec.

### 3.3. Quantitative Analysis of Hypnosis-Related Responses

#### 3.3.1. Subjective Perception of Anxiety, Pain, and Situational Relaxation

Statistically significant differences with large effect sizes were observed between T1 and T2 scores for all three targets: current relaxation, anxiety and pain (see Table 3 and Figure 5). Relaxation scores increased significantly following hypnosis, from M = 5.66 (SD = 1.82) to M = 8.80 (SD = 0.96), an absolute increase of 3.14 points on the NRS (55.5%), W = 0, *p* < 0.001, r = 0.88, a large effect. Anxiety scores decreased significantly following hypnosis, from M = 3.28 (SD = 2.42) to M = 0.88 (SD = 1.16), an absolute decrease of 2.40 points on the NRS (73.2%), W = 105, *p* < 0.001, r = 0.83, a large effect. Pain scores decreased significantly following hypnosis, from M = 2.81 (SD = 2.99) to M = 1.31 (SD = 1.74), an absolute decrease of 1.50 points on the NRS (53.3%), W = 63.5, *p* = 0.004, r = 0.67, a large effect.

#### 3.3.2. Physiological Activity

Due to signal dropouts related to the equipment and a limited number of adequate recordings, the analysis of the level of consciousness (BIS) during the hypnosis exercise could not be conducted. These technical problems are possibly due to the fact that this tool was originally validated under general anesthesia, where sedation levels are considerably deeper. Future studies would therefore benefit from using alternative equipment for this type of measurement in conscious subjects or subjects under hypnosis.

The evolution curves in time of Heart Rate (HR), Breathing Rate (BR), and Nociceptive level (NOL) are presented in Figure 6. As specified in Section 2, time 1 to time 5 denote five consecutive segments of equal duration within each recording and not specific phases of the hypnotic protocol. The linear mixed model analysis first revealed a cubic effect of time on the HR component (β = 56.17; SE = 25.08; *p* = 0.04; f^2^ = 0.39). While the heart rate increased between time 1 (68.86) and time 2 (69.27), it decreased during the hypnosis work between time 3 (69.28) and time 4 (68.95), before increasing again at the end of the procedure (70.51). The analysis also revealed a quadratic effect of time on the BR (β = 145.59; SE = 37.98; *p* = 0.002; f^2^ = 1.22) and the NOL (β = 306.10; SE = 75.13; *p* = 0.001; f^2^ = 1.51), revealing a decrease in breathing rate and nociceptive level from time 1 (BR = 16.25; NOL = 14.92) to time 3 (BR = 14.94; NOL = 12.11), and then a subsequent increase between time 3 and the end of the session (time 5, BR = 17.77; NOL = 17.69). No further effects were found.

## 4. Discussion

This study aimed to define and refine the HYlaMI self-hypnosis training program through individual interviews with individuals suffering from migraines, as well as to evaluate the preliminary effects of a hypnosis exercise from the program (phases Ia & Ib of the ORBIT model). The results highlight four main themes derived from the verbatims of the semi-structured interviews, as well as the effects of a single hypnosis exercise on self-reported relaxation, pain and anxiety, alongside concomitant changes in physiological activity (heart rate, breathing rate, and nociception). Based on these results, improvements were made to the program and recommendations for future interventions are provided.

The first theme addressed ***knowledge and impressions regarding hypnosis***. During the session, participants reported both physical and mental benefits (e.g., pain reduction, decreased anxiety). One participant experiencing a migraine attack reported complete elimination of their pain during the exercise.

**Exploratory Quantitative Analysis.** Physiological activity allowed us to observe changes in respiratory rate, heart rate, and nociception during the *Pleasant Place* hypnosis exercise. Respiratory rate, heart rate and nociceptive level all decreased during the central period of the exercise and returned gradually towards baseline as participants exited the hypnotic state. This pattern is compatible with a transient shift towards parasympathetic predominance, and is consistent with the psychophysiological literature on hypnosis, in which reductions in sympathetic activity and increases in parasympathetic tone are the most frequently reported findings, although effect sizes are heterogeneous and most available data come from small samples of healthy volunteers [58,59].

This relaxation was also reported subjectively by our participants via NRS, which showed a marked increase in relaxation as well as a decrease in pain perception and anxiety following the hypnosis exercise. These findings are consistent with the literature documenting the analgesic and anxiolytic effects of hypnosis, which may be sustained over time [60,61,62,63]. Neuroimaging studies link these alterations in pain perception to changes in pain networks and other brain regions [64]. Nevertheless, our results must be interpreted with caution given the small sample size, which limits statistical power and increases the risk of false positives. Furthermore, in the absence of a control group, the observed effects could also be explained by the passage of time, a practice effect on the measurement instruments, or regression to the mean.

The magnitude of these within-session changes is comparable to that reported after a single session of the HYlaDO program in patients with chronic pain, where pain and anxiety decreased and relaxation increased immediately after the session [27]. This convergence across two clinically distinct populations suggests that the immediate relaxation and analgesia produced by a first, non-specific hypnosis exercise depend only loosely on the pain condition targeted, and that they are therefore poor predictors of the clinical benefit of a complete program. Recommendations for the hypnotic treatment of chronic pain make the same point: response to a single session is a weak proxy for treatment response, and repeated practice is the active ingredient [65]. The same dissociation has been documented for other mind-body approaches to migraine [6,66] Two mindfulness trials in migraine report effects on psychological and burden-related outcomes that do not track effects on attack frequency [6,66]. Taken together, these results argue for combining attack-related endpoints with psychological and burden-related endpoints when HYlaMI enters Phase II, and they support the addition of the Pain Self-Efficacy Questionnaire (PSEQ) described below.

Overall, hypnosis was favourably perceived by the individuals living with migraine who took part in this study. Within the session, it was associated with an increase in relaxation and with reductions in two of the factors that contribute most to migraine-related disability, namely current pain and anxiety.

A second theme explored during interviews was *the **relevance and acceptability of the program***. It enabled the identification of nine concrete avenues for program improvement, as well as suggestions for future interventional studies (see Box 1 and Box 2).

Box 1 **Adaptations made to the HYlaMI program, based on participant recommendations.**

**Adaptations made to the program**

1. Integration of a follow-up session one month after the end of the intervention to reinforce self-hypnosis practice and foster a sense of community;
2. Identification of the time slot for conducting the intervention group (between 10:00 and 12:00);
3. Integration of questions exploring at which phase of migraine (prodromal, ictal, or postdromal) participants would use hypnosis, to better understand when the tool is perceived as most relevant or effective;
4. Integration of measures complementing the questionnaires assessing migraine-related burden, by incorporating measures of work or school absenteeism (participant diary), quality of life (EQ-5D), and the economic burden of migraine (in-house questionnaire);
5. Integration of a psychological outcome (PSEQ) to address a central component linked to migraine-related disability, through the addition of a questionnaire;
6. Improvement of the *Thermal Helmet* exercise, now renamed the *Thermal Object* (visualization of an object with thermal and comforting properties), so that it can be applied to any painful or targeted area of the body.
7. Integration of references so that participants can deepen their knowledge of self-hypnosis as needed;
8. Improvement and integration of certain hypnotic suggestions better adapted to the reality of individuals living with migraine:
  - Removal of references to family;
  - Addition of pain-validating elements so as not to categorically eliminate pain, recognizing that certain forms of discomfort are part of these individuals’ identity and are important to preserve;
  - Addition of suggestions indicating that the unconscious can monitor on the person’s behalf (in relation to relaxation, sleep, and the onset of migraines);
  - Prioritization of neutral sensory language when describing sensory elements, to avoid referencing migraine triggers;
  - Incorporation of a staircase descent during certain deepening phases to assist individuals who experience anticipatory anxiety during a countdown;
  - Addition of suggestions integrating water as an element capable of enveloping and dissolving pain and apprehension;
  - Reminding participants of the relevance of adapting their environment to their needs during sessions, (e.g., suggesting they position themselves in a darkened environment, lie on their back, and apply a cold cloth to their forehead, eyes, or the back of the neck);
  - Addition of a note clarifying that having multiple mental images simultaneously is appropriate and that there is no need to try to control them.
9. Development of a clinician and participant manual to further standardize the intervention.

Box 2 **Suggestions for future hypnosis interventional studies for migraine.**

**Possible Interventional Levers for Migraine**

*Psychological therapeutic targets:*

Anticipatory anxiety, performance anxiety, depression, guilt related to loss of functioning, and discouragement in the face of therapeutic failures.

*Kinesthetic therapeutic targets:*

Post-attack recovery, aimed at re-energizing the body following a migraine episode, and targeting physical hypersensitivities to reduce sensory irritability during an attack.

*Psychoeducation:*

On energy management, particularly the importance of pacing oneself on good days to better cope with difficult periods.

**Elements to Explore in Future Research Protocols**

*Migraine phase:*

Examining the effect of hypnosis at different stages of migraine (e.g., prodromal, ictal, postdromal).

*Group composition:*

Forming groups based on similar pain or migraine profiles to encourage more targeted exchanges.

*Incorporating catch-up sessions:*

Planning catch-up sessions for individuals who missed a session due to a migraine attack.

**Elements to Consider**

*During in-person sessions:*

Taking sensory hypersensitivities into account during in-person sessions (e.g., dimmed lighting environment).

*During hypnosis exercises for migraine:*

Addressing pain in a holistic manner and not limiting the focus to the cephalic region.

**Program format.** Overall, participants had a favorable impression of the program, describing it as relevant, well-structured, and well-suited to the challenges of migraine, and all expressed interest in participating. The program format, consisting of eight weekly 1-h group sessions delivered virtually to groups of 8–9 participants, with each session incorporating 20–30 min of formal permissive hypnosis and audio recordings provided to support self-hypnosis practice at home, was deemed appropriate. Eight weekly one-hour sessions combining practitioner-guided hypnosis with recorded home practice is also the structure adopted by HYlaDO for chronic pain, and group hypnosis protocols delivered in outpatient integrative medicine typically comprise six to eight weekly sessions of 60 to 90 min with audio-supported home practice; the present format is therefore consistent with that of recent hypnosis interventions [27,43,67] and recommendations for hypnotic treatment of chronic pain [65].

The option to follow the program remotely was perceived as a major asset in terms of accessibility and flexibility, as it may mitigate several of the drawbacks associated with in-person group interventions, advantages long noted in previous online non-pharmacological migraine interventions (e.g., cognitive-behavioral therapy) [68,69]. However, questions were raised regarding the equivalence in efficacy of in-person versus telepractice, as well as the social needs of individuals with migraine. A recent international post-COVID-19 survey of clinicians using hypnosis found that nearly two-thirds of respondents (63.8%) reported using hypnosis via telepractice, and more than half (54%) indicated it was just as effective as face-to-face practice [70]. This modality also offers several advantages over in-person delivery (e.g., accessibility, reduced costs, decreased transportation-related anxiety) [71]. Hypnosis telepractice therefore appears appropriate for this population, combining both efficacy and accessibility.

To address the social need mentioned by several participants, a one-month follow-up session will be added to the program HYlaMI, allowing them to share their experiences and encourage continued self-hypnosis practice (see Box 1).

**Optimal timing.** Beyond format, participants were asked about the ideal time of day to administer the intervention and self-hypnosis. Although the 10:00–12:00 window emerged as the window during which this sample of the migraine population generally felt most functional, no clear consensus emerged, reflecting the variability in migraine profiles across participants. This time slot will therefore be prioritized for the continuation of the HYlaMI program (see Box 1). However, whether it remains the preferred window for future participants remains to be seen, as migraine chronobiology research tends to indicate a higher risk of attacks during this period [10]. One interesting way to address this question comes from a recent pilot cross-over randomized trial on exercise interventions for migraine, which found that aligning exercise timing with participants’ individual chronotype enhanced the efficacy of the exercise prescription [72]. Building on this, future non-pharmacological interventions could explore whether synchronizing intervention timing with participants’ chronotype similarly improves adherence and efficacy.

**Therapeutic objectives.** Participants endorsed the therapeutic objectives of the intervention but questioned the feasibility of migraine-attributed burden as a primary outcome, given the variability of lived experience across migraine profiles.

To address this concern and broaden the dimensions captured in the measurement of migraine burden, our study incorporates the two questionnaires assessing migraine-related disability recommended by the International Headache Society (HIT-6, MIDAS), to give a more accurate assessment of a participant’s headache-related disability [34,73]. We will also be including measures of work or school absenteeism, quality of life (EQ-5D) [74] and the economic burden of migraine. Cost data are increasingly requested by payers and decision-makers to arbitrate the funding of non-pharmacological programs, and their near-absence in the hypnosis literature is a recognized obstacle to implementation; collecting them from Phase II onwards avoids having to reconstruct them retrospectively at the trial stage [75]. Lastly, the PSEQ [76] will be added to the protocol. This psychological measure has been used in other non-pharmacological interventions for chronic migraine [77,78] and aligns with one of our study’s goals: empowering participants and building their confidence to implement self-management strategies, including self-hypnosis, to manage their migraines and associated symptoms (see Box 1).

**Session themes and content.** Participants appreciated the hypnosis exercise themes proposed in the program and their comprehensiveness, as well as the content of the *Pleasant Place* exercise experienced during the session. Their comments aligned with our recent literature review, which recommends integrating diversified hypnosis exercises (e.g., centered on pain perception and the development of internal resources), in order to cover a wider range of facets of people living with migraine’s reality [22]. As a concrete adjustment to the program, the *Thermal Helmet* exercise has been modified for the *Thermal Object*, allowing comfort to be directed to any area of the body based on individual needs, and a reference resource on self-hypnosis will be recommended as a supplement. The hypnotic suggestions themselves were also revised in nine respects, listed in full in Box 1. The programme’s existing combination of kinesthetic and visualization exercises accommodates individuals less comfortable with imagery, and the Ericksonian permissive approach allows continual adaptation to individual experience within the group [79]. Additional recommendations were put forward (see Box 2; e.g., ideas for therapeutic themes), but cannot be fully incorporated into the program, as the number of sessions is already fixed at eight and all existing exercises were unanimously well-received.

**Who can deliver the intervention.** Beyond content, participants shared their views on the desirable profile of the practitioner: in their view, the intervention could be delivered by any healthcare professional with training in hypnosis and the requisite knowledge, a pleasant tone of voice, and basic relational qualities such as compassion and attentive listening. These characteristics are consistent with those outlined by the International Society of Hypnosis (ISH) [80].

The third theme was ***expected and perceived effects of the program***, detailed in Section 3.2. Participants viewed hypnosis as a complementary treatment alongside pharmacological therapies, with the potential to gradually reduce medication use. According to our recent systematic review, hypnosis may be associated with improvements across several of these factors (e.g., migraine frequency, intensity, and duration, psychological factors, and medication use) [22]. This review also highlights the considerable methodological heterogeneity and lack of standardization in the literature, underscoring the need to develop new hypnosis interventions for migraine management. To that end, the HYlaMI program will be fully manualized before entering the next stage of development (see Box 1).

The fourth theme was ***unanticipated elements***. Several participants spontaneously raised considerations relevant to working with this clinical population, particularly the stigma they experience in their daily lives and the need for greater integration of non-pharmacological approaches into conventional medical care.

**Stigma and migraine.** First, the interviews revealed a profound sense of being misunderstood among individuals with migraine, both by their social environment and the medical system. The trivialization and general lack of awareness surrounding migraine contribute to the isolation of many affected individuals, a phenomenon that, according to some participants, is rooted in a history of gender-based stigmatization that has long hindered research and the recognition of effective treatments. Migraine-related stigma extends well beyond gender, affecting the cultural, structural, medical and occupational spheres alike, and leading to an internalized stigma detrimental to self-esteem [81,82]. A recent US population-based analysis highlighted that this stigma still affects 25% of individuals with episodic migraine and 47% of those with chronic migraine, worsening the associated disability, burden, and quality of life [83], while durably undermining the social acceptability, recognition, and management of the condition [81,84]. These stigmas must be considered when working with this population and actively addressed, through public and patient education and clinician training in migraine, among other approaches [81].

**Toward an integrative approach.** Participants also expressed weariness with a medical approach that is almost exclusively pharmacological, advocating for greater dialogue between neurologists and complementary medicine practitioners. The intervention HYlaMI was thus perceived as a welcome response to a self-management need that is insufficiently addressed by conventional medicine, and several participants emphasized the importance of broadening its reach. Indeed, only one-third of individuals with migraine report being satisfied with their treatment, leading many to turn to complementary approaches [85], underscoring the need for a holistic approach to migraine management that integrates both pharmacological and non-pharmacological strategies.

**Positioning HYlaMI among non-pharmacological approaches to migraine.** The acceptability profile observed here is best read against that of the other non-pharmacological options currently available to people with migraine. Mindfulness-based programs are the most extensively evaluated: they are well received and improve migraine-related disability, quality of life, self-efficacy and pain catastrophizing, but they are demanding, and feasibility work reports adherence close to two-thirds for a format of eight weekly two-hour classes plus a day-long retreat [6,86]. Biofeedback reduces headache frequency and severity relative to waiting-list control, though not relative to active treatments, and requires equipment and repeated supervised sessions [7]. Aerobic exercise and yoga improve attack frequency and intensity but are difficult to sustain during the prodromal and ictal phases, precisely the moments at which our participants judged an intervention most useful [83]. Seen in this light, the features our participants valued most in HYlaMI—one-hour rather than two-hour sessions, remote delivery, and a portable skill deployable during an attack without equipment—address recognized barriers to the existing non-pharmacological offer rather than duplicating it.

Compared with HYlaDO, the chronic pain program from which HYlaMI was derived, social acceptability was at least as high in the present sample. Participants in the HYlaDO qualitative studies similarly valued the teleconsultation format, the take-home audio recordings and the progressive structure of the exercises, and similarly framed hypnosis as a complement to, rather than a substitute for, medical treatment [26,28]. Two differences appear specific to migraine. First, our participants insisted that the exercises address the neck and shoulders and not only the cephalic region, and that sensory suggestions avoid known attack triggers—constraints that do not arise in undifferentiated chronic pain, and which led us to replace the *Thermal Helmet* by the Thermal Object. Second, relaxation itself was ambivalent for two participants who associate it with the nocturnal onset of their attacks, a phenomenon we had not encountered in chronic pain samples, and which required adding suggestions that explicitly preserve a form of unconscious vigilance. These observations suggest that transferring a generic hypnosis program to a new pain condition requires more than substituting the content of the exercises: the very meaning of core hypnotic targets such as relaxation may differ across conditions and should be probed before rather than after a program is deployed.

### Limitations

The limitations below should be read against the criteria applicable to this phase of development [32,33]. ORBIT Phase Ib is a refinement phase, whose standards of evidence are those of intervention development rather than of a hypothesis-testing trial. The absence of a control condition, the small purposive sample and the non-probabilistic recruitment are design features of this phase, addressed in Phases IIa and IIb; they bound the conclusions to the acceptability and refinement objectives, and no claim of efficacy or generalisability is made. A first limitation is that the same individual (ÉC) conducted the recruitment, interviews, administration of the hypnosis session, and both the qualitative and quantitative analyses (NRS only). While this may introduce bias into our analyses, this choice was made deliberately to allow for a deeper understanding of the data emerging from the qualitative analysis. We also sought to mitigate its impact through inter-rater agreement and verification of the quantitative analyses by other team members. A second limitation is that participants completed the NRS verbally with the intervener, which may have introduced social desirability bias. A third limitation is that the physiological activity monitoring was not segmented in accordance with the distinct phases of hypnosis or specific suggestions, thereby restricting the interpretation of the results. Because the five time segments were obtained by dividing each recording into equal parts rather than by aligning it with protocol events, segments of the same rank may correspond to different hypnotic phases across participants—some participants were already engaged in the hypnotic work at time 2 while others were still in the deepening phase. The observed curvatures therefore describe the overall temporal evolution of each signal and cannot be attributed to specific suggestions. Addressing this limitation in future studies will allow for a more nuanced understanding of the underlying mechanisms of hypnosis in migraine. A fourth limitation is the absence of a control condition: the within-session changes cannot be attributed to the exercise itself, since the passage of time, the resting position, the attention of the interviewer, expectancy and regression to the mean remain plausible contributors, and they are reported as signal detection rather than as evidence of efficacy. A fifth concerns the self-report measures: single-item scales admit no internal-consistency coefficient and have no population-specific psychometric data in migraine, so their reliability and validity here are unestablished. They were chosen for their brevity and their sensitivity to rapid change. Finally, a last limitation concerns the sample and its size. Indeed, the small sample and its demographic characteristics (e.g., predominantly Caucasian women aged 55 living in Quebec, Canada) limit the statistical power and the generalizability of this study. However, limited generalizability is a common challenge in clinical research, particularly given that women tend to be more likely than men to participate in research studies. Moreover, the predominance of women in the present sample is consistent with the well-documented sex difference in migraine prevalence, with migraine being substantially more prevalent among women than men. Nevertheless, the limited representation of men warrants caution when extending these findings to male patients with migraine, all the more so as the sex difference in prevalence noted in the Introduction is expected to narrow rather than disappear. Three further considerations temper the interpretation of the acceptability data. First, participants were recruited through a patient organization and volunteered for a study explicitly presented as involving hypnosis, which plausibly selected individuals already favourable to non-pharmacological approaches and inflates the enthusiasm reported here; acceptability in an unselected clinical population may well be lower. Second, hypnotic suggestibility was not assessed, although it moderates the response to hypnotic analgesia; including it from Phase II onwards would allow the immediate responses observed here to be interpreted in relation to individual hypnotic capacity. This was deliberate: a standardised susceptibility scale would have added about an hour to a 90-min session and, being itself a hypnotic procedure, would have given every participant a prior experience of hypnosis. Hypnotisability nevertheless modulates both the experience of hypnosis and its cortical correlates and will be assessed from Phase IIa as a moderator of response. Third, the presence of other chronic pain conditions was not documented systematically, so the contribution of non-migrainous pain to the reported daily pain levels cannot be established. The baseline daily pain level should therefore be read as an index of overall pain burden rather than as a migraine-specific quantity. A systematic inventory of comorbid pain conditions is planned for Phase II.

## 5. Conclusions

In conclusion, the present study supports the social validity of HYlaMI and provides insights for the development and optimization of hypnosis-based interventions for migraine. Social validity here denotes the extent to which the goals, procedures and effects of a program are judged acceptable and worthwhile by the people it is intended for. Participants expressed strong interest in both hypnosis and the program, highlighting the potential value of hypnosis as a non-pharmacological approach to complement standard migraine care. Their feedback informed the refinement of the intervention and identified key considerations for its future implementation and other studies. In line with the ORBIT model, the next step (Phase II) will be a proof-of-concept and pilot study to examine the preliminary effects and feasibility of the refined intervention, providing a foundation for subsequent randomized controlled trials in Phases III and IV.

Beyond HYlaMI, these findings support the acceptability of structured, manualized training programs based on hypnosis as a complement to conventional migraine management, and the preliminary within-session data justify pursuing their development. Three findings were not predictable from the chronic pain literature: the ambivalence of relaxation for participants who associate it with the nocturnal onset of their attacks, the need to address the neck and shoulders and not only the cephalic region, and the need for sensory suggestions to avoid recognised attack triggers. Each led to a concrete modification of the program (Box 1).

Two lessons extend to other teams. The content of hypnotic suggestions must be adapted to the specific phenomenology of the target condition rather than transferred wholesale from generic chronic pain protocols, since core targets such as relaxation may carry a different, and occasionally adverse, meaning for the population concerned. And outcome selection in early-phase work should pair migraine-attributed burden with psychological mechanisms such as pain self-efficacy, which is where mind-body interventions for migraine have most consistently demonstrated benefit.

## Figures and Tables

**Figure 1:**
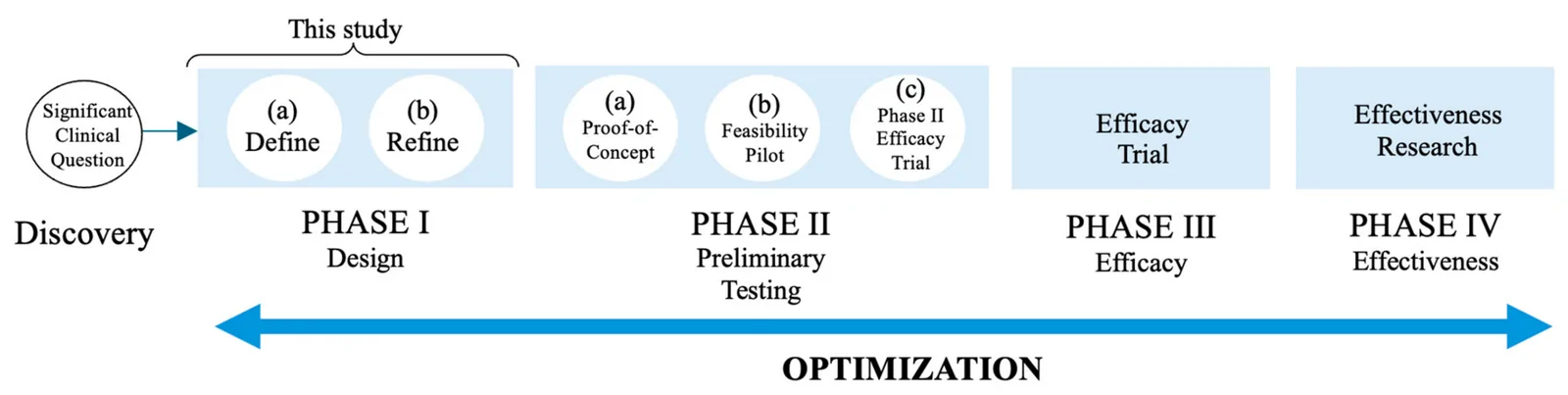
The ORBIT Model for Behavioral Treatment Development.

**Figure 2:**
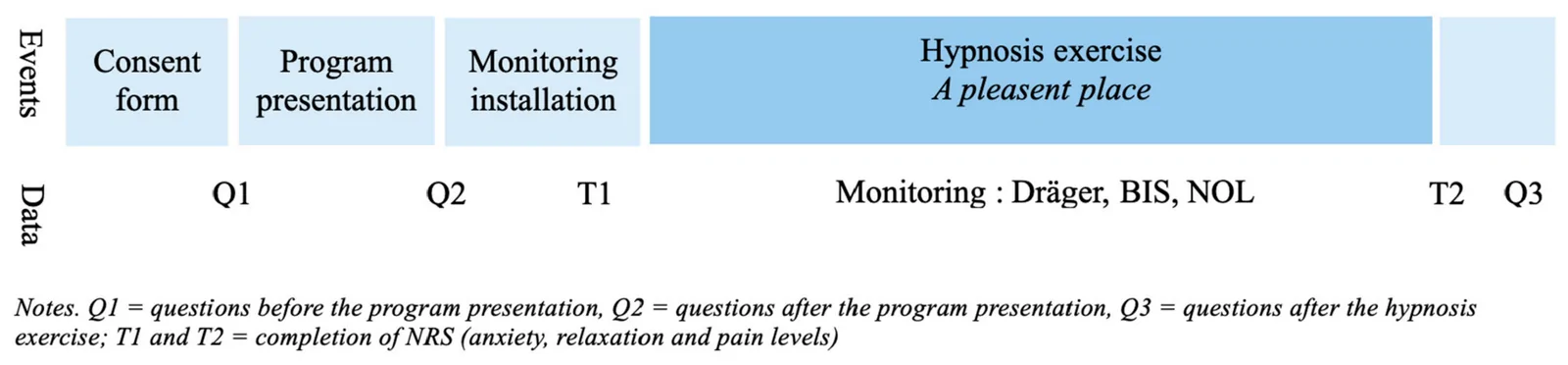
Research protocol.

**Figure 3:**
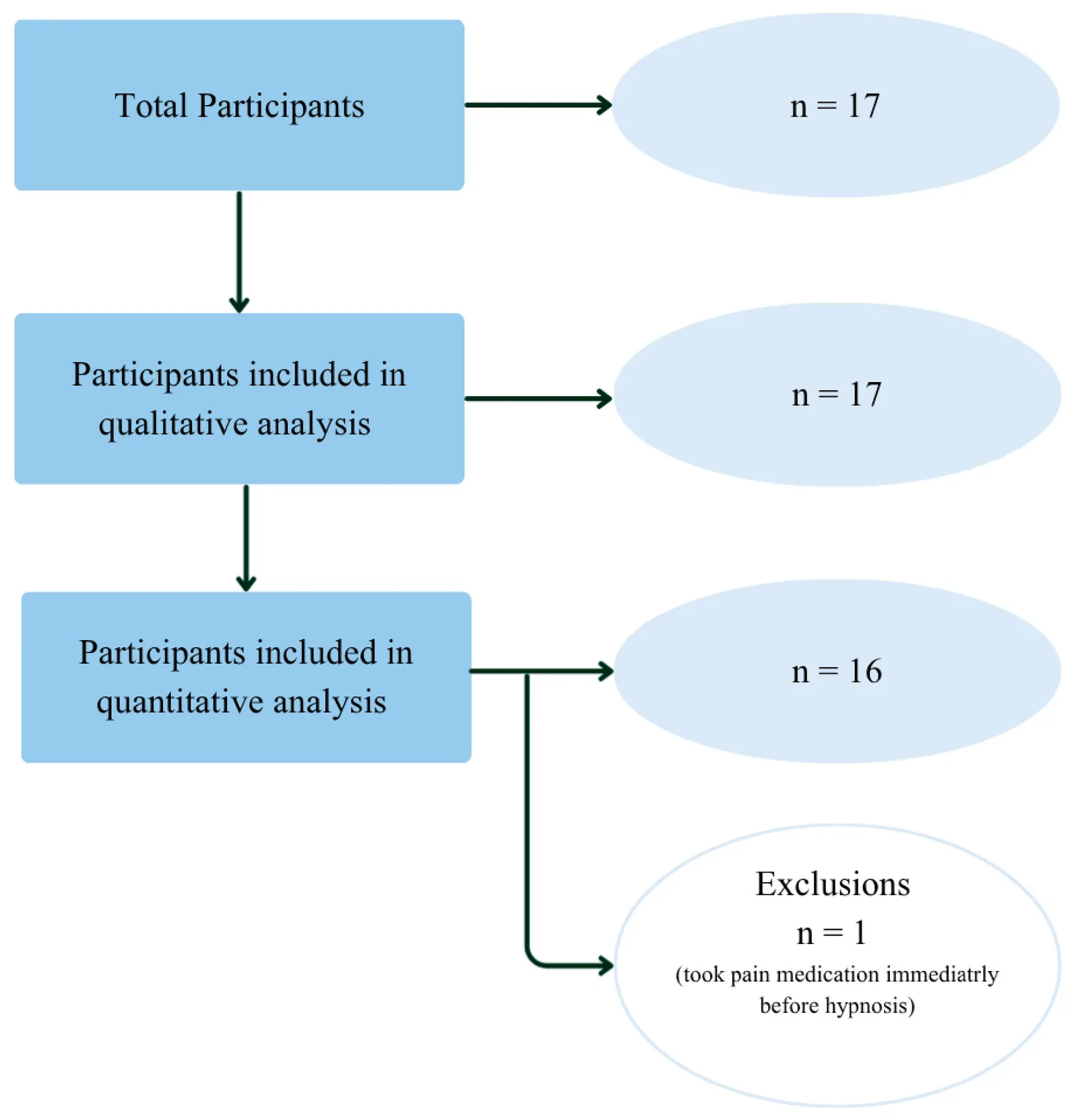
Participant Flow Diagram.

**Figure 4:**
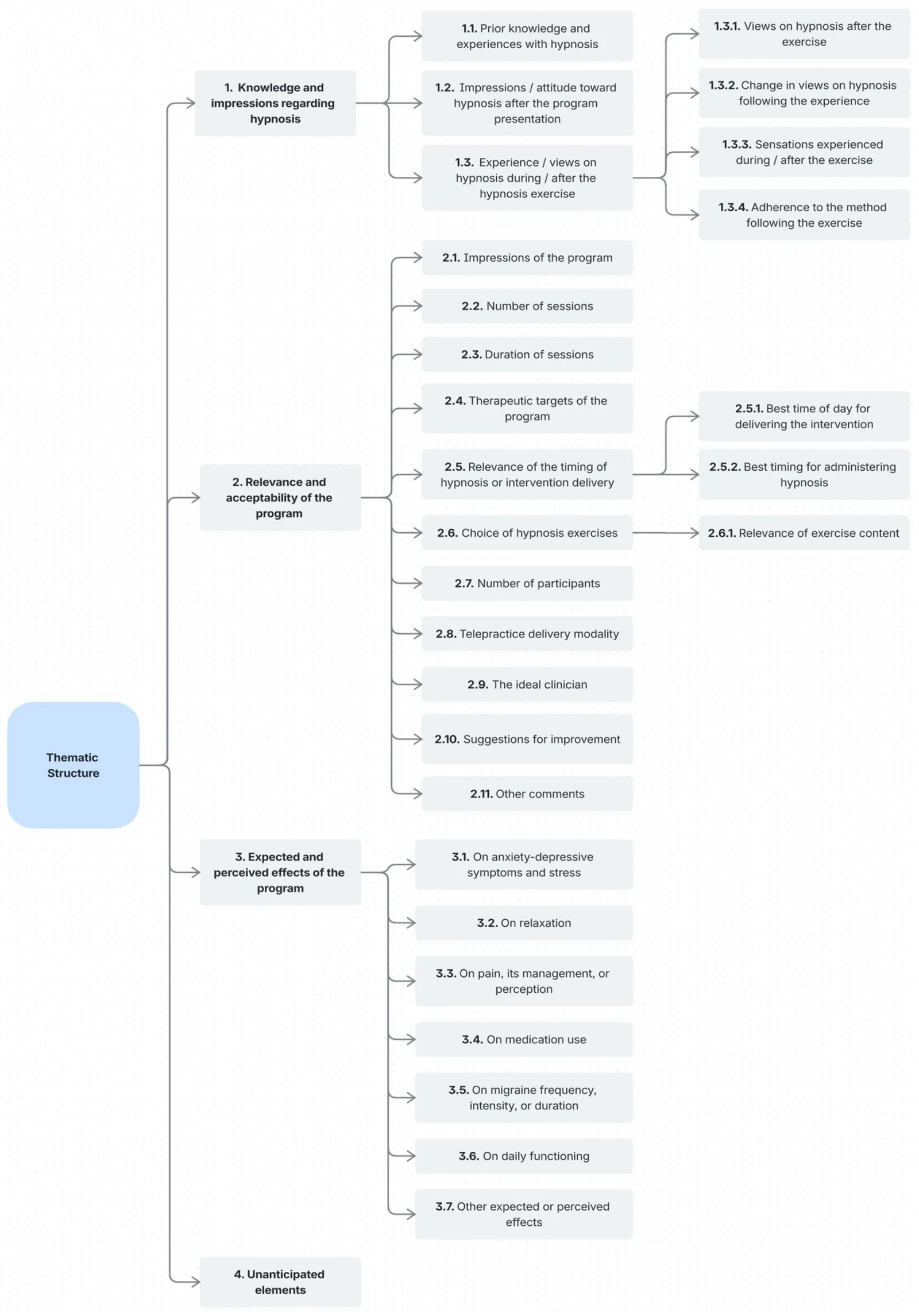
Main Themes and Sub-Themes Derived from Interview Thematic Analysis.

**Figure 5:**
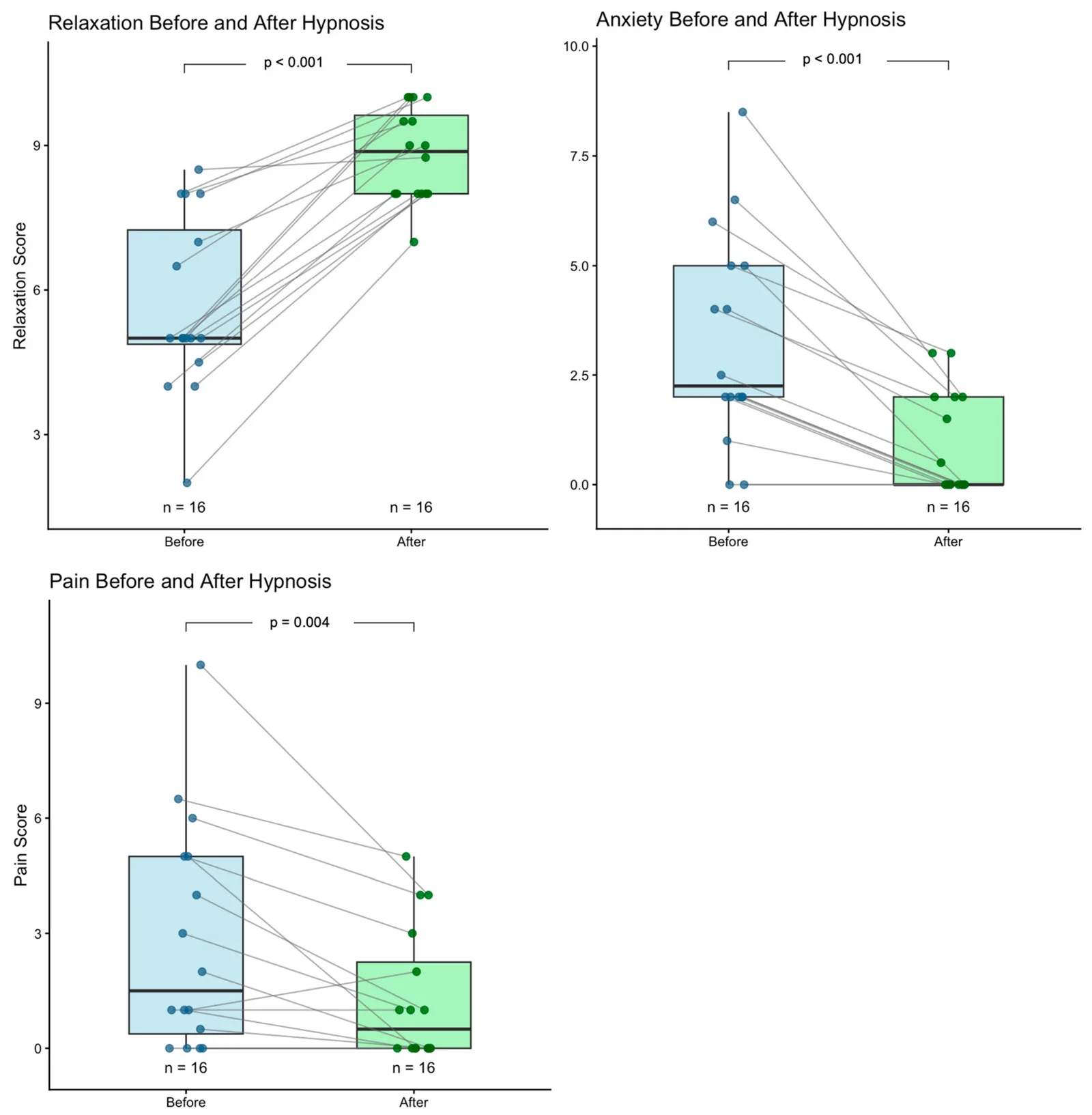
Changes in self-reported relaxation, anxiety and pain 11-point numeric rating scales (0–10) before (T1—in blue) and after (T2—in green) the hypnosis exercise (*n* = 16). Boxes show the median and interquartile range, points the individual scores, and grey lines connect each participant’s pre- and post-exercise scores.

**Figure 6:**
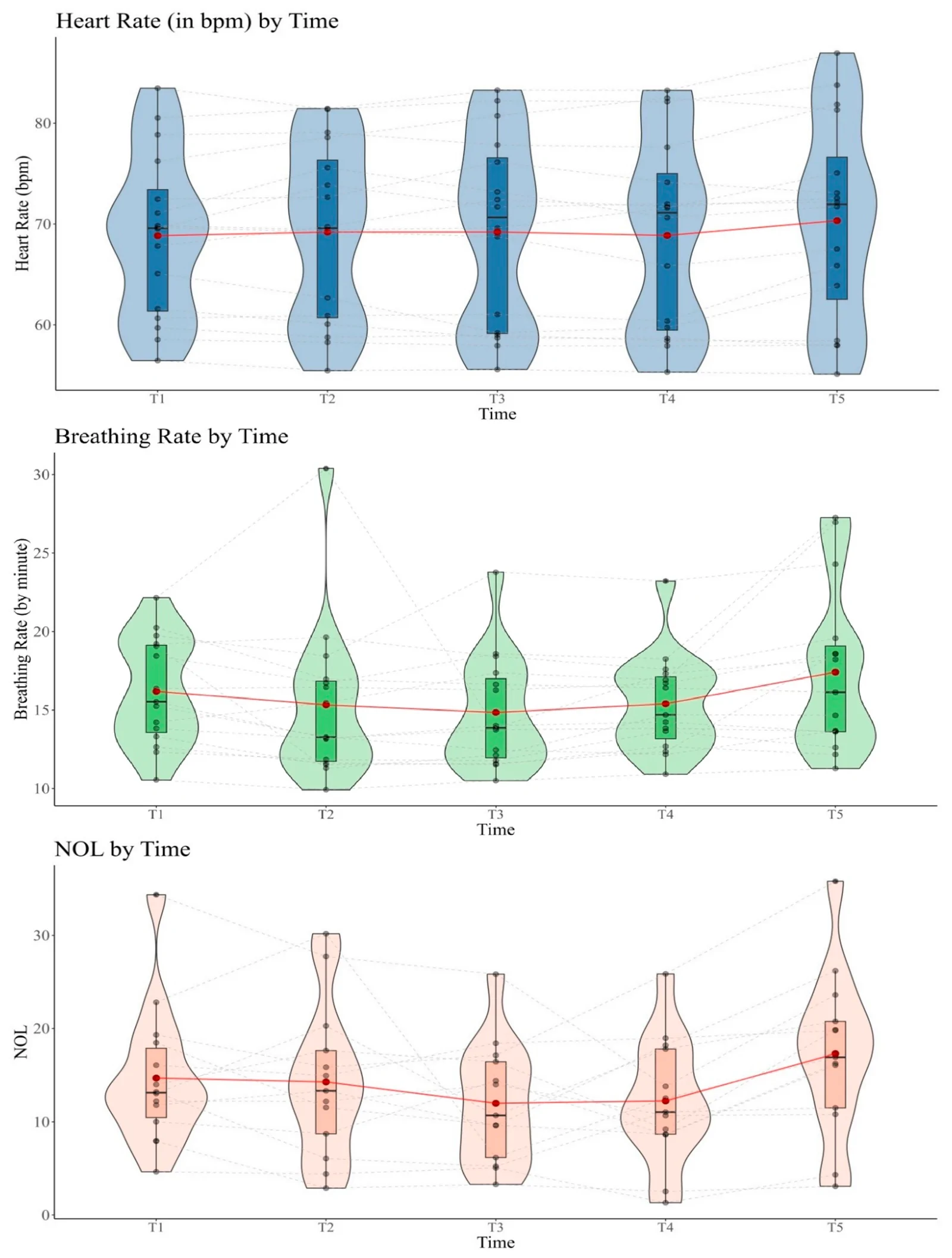
Evolution curves of Heart Rate (HR), Breathing Rate (BR), and Nociceptive Level (NOL) depending on Time. Time 1 to time 5 are five consecutive segments of equal duration within each recording, not phases of the hypnotic protocol (*n* = 16; *n* = 15 for BR). Violin and box plots show the distribution within each segment, grey dashed lines the individual participants, and the red line the segment means.

**Table 1:** Descriptive Analysis of the Sample (*N* = 17).

Features		Number of Participants or Distribution of Features
Sex (gender), *n*	Woman (she)	15
	Man (him)	2
Age	Mean	55.88
	Minimum	32.00
	Maximum	71.00
	Median	51.00
	SD	12.11
Ethnic Affiliation, *n*	Caucasian	16
	Latin American	1
Marital status, *n*	Married	8
	In a relationship	5
	Divorced or separated	2
	Single	2
Number of Children, *n*	0	5
	1	3
	2	7
	3	2
Education, *n*	High school	1
	College	6
	University	10
Professional status, *n*	Full-time	2
	Half-time	1
	Retired	5
	Work disability	7
	Unemployed (student)	1
	Temporary leave from work	1
Migraine type (self-reported), *n*	Episodic	4
	Chronic	10
	Menstrual	1
	Vestibular	1
	Other	1
Time since migraine onset (years)	Mean	22.35
	Minimum	2.00
	Maximum	60.00
	Median	20.00
	SD	16.73
Familial migraine history, *n*	Yes	12
	No	5
Frequency of migraine days (/30 days)	Mean	13.75
	Minimum	1.00
	Maximum	30.00
	Median	12.00
	SD	10.26
Medication	See Supplementary Material Table SM4	
Psychological diagnosis, *n*	Yes	8
	No	9
Type (when mentioned), *n*	Anxiety	4
	Depression	1
	PTSD	1
	CPTSD	1
Usual daily pain, all causes combined (NRS 0–10)	Mean	6.56
	Minimum	2.50
	Maximum	10.00
	Median	6.50
	SD	1.75
Usual daily anxiety (NRS 0–10) *(n = 16; one NA)*	Mean	5.28
	Minimum	1.00
	Maximum	8.00
	Median	5.75
	SD	2.29

Note. *N* = 17 unless otherwise specified. SD = standard deviation; NRS = numeric rating scale scored from 0 to 10, administered verbally; PTSD = post-traumatic stress disorder; CPTSD = complex post-traumatic stress disorder. All clinical variables were self-reported by participants and were not verified against medical records; chronic migraine follows the ICHD-3 definition [48]. Usual daily pain and usual daily anxiety refer to the participant’s typical daily state, all health conditions combined. The complete list of medications reported by participants, using international non-proprietary names, is provided in Supplementary Material Table SM4.

**Table 2:** Summary of the Four Themes.

Themes	Summary
Theme 1. Knowledge and impressions regarding hypnosis	Most participants approached hypnosis with limited knowledge, often drawn from media or stage shows. Some initially expressed a need for scientific legitimacy before trusting the method. The program presentation helped address these reservations, making the intervention more concrete and confirming the positive intuitions many participants already held. The first hypnosis exercise was generally experienced positively: participants described deep relaxation, a shift in their perception of pain, inner calm, and vivid mental sensations. All expressed, by the end of the session, a strong desire to continue and integrate self-hypnosis as an autonomous management tool.
Theme 2. Relevance and acceptability of the program	Participants responded to the program in an overwhelmingly positive way, describing it as well-structured, relevant, and well-suited to the reality of people living with migraines. The format of eight one-hour weekly sessions was considered appropriate, and the telepractice delivery was seen as a major asset for accessibility. The therapeutic targets, such as functional disability and psychological factors, were unanimously validated. The proposed exercises, particularly the magic hand and the pleasant place, were especially appreciated. Participants also offered several suggestions for improvement, including some personalization of hypnotic suggestions, the addition of post-crisis recovery sessions, follow-up meetings, and access to supplementary resources.
Theme 3. Expected and perceived effects of the program	Participants anticipated benefits across multiple dimensions. Reduction of stress and anxiety was the most widely expected effect, with downstream impacts on mood, motivation, and daily functioning. The intervention was also perceived as an effective tool for physical and mental relaxation, particularly helpful for recovery after migraine episodes. Regarding pain, participants expected an improvement in their ability to tolerate and cope with it daily, rather than a complete cure. Reduced medication use was also frequently mentioned, with hypnosis envisioned as a non-pharmacological complement to existing treatments. Finally, indirect effects on migraine frequency and intensity were anticipated, along with an overall improvement in quality of life.
Theme 4. Unanticipated elements	Some participants spontaneously expressed a deep sense of being misunderstood, both by those around them and by the medical system. They expressed that migraines are frequently trivialized, leaving many feeling isolated with their condition. They linked this lack of understanding with a history of gendered stigmatization, as the condition’s prevalence among women has long limited research and treatment recognition. Participants also voiced weariness toward a medical approach centered almost exclusively on medication, calling for greater dialogue between neurologists and complementary care providers. In this context, the intervention was seen as a welcome response to a self-management need that traditional medicine has yet to fully address.

**Table 3:** Descriptive Statistics for the Self-Reported Outcome Variables (*n* = 16).

	R-T1	R-T2	A-T1	A-T2	P-T1	P-T2
N	16	16	16	16	16	16
M	5.66	8.80	3.28	0.88	2.81	1.31
Mdn	5.00	8.88	2.25	0.00	1.50	0.50
SD	1.82	0.96	2.42	1.16	2.99	1.74
Range	6.50	3.00	8.50	3.00	10.00	5.00
Min	2.00	7.00	0.00	0.00	0.00	0.00
Max	8.50	10.00	8.50	3.00	10.00	5.00

Note. R = Relaxation; A = Anxiety; P = Pain; T1 = before hypnosis; T2 = after hypnosis. M = Mean; Mdn = Median; SD = Standard deviation. One of the 17 participants was excluded from the quantitative analyses (see Figure 3). Scores are expressed in points on 11-point NRS (0–10), administered verbally by the interviewer. T1 and T2 measures taken immediately before and after the hypnosis exercise are distinct from usual daily pain and usual daily anxiety referring to the participant’s typical daily state, all health conditions combined (Table 3).

## Data Availability

The data presented in this study are not publicly available due to privacy and ethical restrictions. The dataset consists of qualitative interview transcripts and individual physiological recordings that could compromise the privacy of participants, who did not consent to public data sharing. De-identified data supporting the conclusions of this article are available on reasonable request from the corresponding author (D.O.), subject to approval by the Research Ethics Board of the CIUSSS de l’Est-de-l’Île-de-Montréal. The original audio recordings were destroyed following transcription, in accordance with the approved research protocol.

## Supplementary Materials

The following supporting information can be downloaded at: https://www.mdpi.com/article/10.3390/brainsci16101007/s1, Supplementary Material File SM1. HYLAMI evaluation grids; Supplementary Material File SM2. Pleasant Place script HYlaMI; Supplementary Material File SM3. Thematic tree (code list); Supplementary Material Table SM4. Medication List; Supplementary Material Table SM5. Verbatims for themes and sub-themes.

## Abbreviations

The following abbreviations are used in this manuscript:

HYlaDO	Hypnosis for Chronic Pain, in French, HYpnose pour le DOuleur chronique
HYlaMI	HYpnosis for Migraine, in French, HYpnose pour la MIgraine
ORBIT	Obesity-Related Behavioral Intervention Trials
HIT-6	Headache Impact Test—6 items
MIDAS	Migraine Disability Assessment
NRS	Numeric rating scale
HADS	Hospital Anxiety and Depression Scale
PSEQ	Pain Self-Efficacy Questionnaire
VAKOG	Hypnosis technique: visual, auditory, kinesthetic, olfactory, gustatory
P1-P17	Participants numbers
T1	Measure before the hypnosis exercise
T2	Measure after the hypnosis exercise
Q1	Questions before the presentation of HYlaMI program
Q2	Questions after the presentation of HYlaMI program
Q3	Questions after the hypnosis exercise
HR	Heart rate
BR	Breathing rate
NOL	Nociceptive level
BIS	Bispectral Index, used here as an index of the level of consciousness
SD	Standard deviation
PTSD	Post-traumatic stress disorder
CPTSD	Complex post-traumatic stress disorder
ISH	International Society of Hypnosis
