# Supplementary material for: Development and Refinement of HYlaMI, a Self-Hypnosis Training Program for Migraine Management: An ORBIT Phase Ia–Ib Acceptability Study
Source: Brain Sci. 2026 Sep 23;16(10):1007. doi: 10.3390/brainsci16101007 (PMC13702207; doi:10.3390/brainsci16101007)
Supplement: Supplementary file 1 [file brainsci-16-01007-s001.zip › brainsci-4535718-supplementary.pdf]

File SM1. HYlaMI Program Evaluation Grids

Development and Refinement of HYlaMI, a Self-Hypnosis Training Program for Migraine Management

** Based on the Semi-Structured Interview for Social Validity Assessment*

[Have participants complete the sociodemographic information form (to be completed in advance) and the informed consent form: 10 minutes]

START audio recording

Q1- INTRODUCTION QUESTIONS

- What do you **know** about hypnosis?

PAUSE audio recording

[PowerPoint presentation of the program; 20 minutes]

RESUME audio recording

Q2- POST-THEORY QUESTIONS

- **Impressions on hypnosis**
  - What is your opinion of the self-hypnosis training program?
  - What is your general opinion of hypnosis?
  - How do you find the program's suitability for a migraine management program?

- **Relevance and acceptability of the program**

  Program structure
  - Does the number of sessions seem adequate?
  - Does the duration of the sessions seem appropriate?
  - Do the therapeutic targets seem appropriate?
  - Would there be a better time of day to deliver the intervention?
  - What do you think of the choice of hypnosis exercises?
  - Are there any themes you would find appropriate, helpful, or relevant to address?
  - Do you think the number of participants is adequate?
  - Do you think remote delivery (telepractice) is appropriate?

  Clinician
  - Who would be an ideal clinician for this program, in your opinion?

Do you have any other comments regarding your impressions of the program?

PAUSE audio recording

** Gradually install monitoring equipment [NOL, BIS, Drager] **

T1- COMPLETE PAIN, ANXIETY, AND RELAXATION QUESTIONNAIRES PRE-HYPNOSIS

** Ensure BIS, NOL, and Drager monitoring equipment installation is complete **

[Hypnosis exercise: The Pleasant Place _ 30 minutes]

RESUME audio recording

Q3- POST-HYPNOSIS EXPERIENCE QUESTIONS

- **Hypnosis experience**
  - What was your experience following the hypnosis session?
  - What do you think of medical hypnosis in the context of migraine management?
  - Did your opinion of hypnosis change following this brief experience? If so, how?
- **Expected and perceived effects**
  - What are the expected vs. perceived effects following your participation in the program?
  - Regarding the reduction of anxiety/stress?
  - Regarding relaxation?
  - Regarding medication use?
  - Regarding daily functioning?
  - Regarding the frequency/intensity/duration of migraines?
    Regarding other expected/perceived effects? Which ones?
  - Any other comments?

- **Recommendations**
  - Do you have any recommendations to improve this program?
  - What should we improve in our program?
  - How should we adapt the techniques to facilitate implementation in a clinical setting?
  - Any other recommendations?

STOP audio recording

T2 - COMPLETE PAIN, ANXIETY, AND RELAXATION QUESTIONNAIRES POST-HYPNOSIS

Total interview duration: approximately 45 minutes

File SM2. Pleasant Place Script - HYlaMI

TIME BREAKDOWN
7:00 the body becomes heavy
9:75 deepening
12:30 vakog
16:40 walk
19:56 deepening #2
21:42 Two minutes of silence
24:30 meditation
26:30 erasing tensions
31:30 2 minutes of silence
33:45 return to the waking state
36:00 end

Induction
You can lie down and get comfortable. I simply invite you to **close your eyes**.
Focus on your **breathing**, letting your belly rise on the inhale, letting the air simply enter through your nostrils. On the exhale, let the unneeded air escape through your mouth or your nostrils.

You can simply let yourself be rocked by this movement of the **breath**, the belly rising on the inhale, falling on the exhale.

And with each movement of inhaling and exhaling, perhaps you can feel the **relaxation** settling in and begin to feel the relaxation of the muscles of your face as well

[starting from the forehead down to the feet].

Perhaps it's as if your eyelids are finally starting to **grow heavy**. Perhaps you can feel the **weight of your body**, feel this body becoming more and more **heavy**. And simply, by **doing nothing**, letting it happen, without resisting, you can let your eyelids close comfortably.

With your eyelids closing, it's a bit like **closing the shutters on the outside world**… to focus more on your inner experience…

[Eyes close]

And now, you don't **really need** to do anything, to look for anything, or to stop anything from happening

I'm simply going to invite you to… **welcome**, to notice everything that happens… thoughts… sensations… distractions… allowing your mind to go wherever it wants… inside or outside…

And you don't really need either… **to look for anything** at all… nor to try to do anything.
In fact, you don't even have to listen carefully to everything I say, because, inevitably, **your unconscious can hear and understand** what is useful and necessary for you…

And you can give yourself full **permission to have whatever kind of experience** suits you… not forgetting to include in your experience… your thoughts… your distractions… even your discomforts…

So that little by little… you can **find your own way of being in a trance**…
And you can continue to be present…

observing and listening to everything I say while continuing to let go… first allowing your body… to relax…

And I don't know whether you'll go into trance… **deeply**… moderately… or lightly… but it doesn't really matter…

And while continuing to hear my voice.
you can simply allow your body…
to be as **deeply relaxed** as it wishes…
And for that, you can take one big, deep breath before slipping… **comfortably relaxed.**

[Step-by-step muscular relaxation]

The idea is simply to allow your body to experience… a **total… complete** muscular relaxation. letting yourself slip into this particular state of **deep relaxation……** into this particular, truly **pleasant… truly enjoyable…** state of mind.

Having a **body free** … of all tension… of all restraint… free of all stress
And while listening to my voice … which guides you into this particular state **… of deep, total …and complete relaxation….**

I invite you to let the **relaxation** now **move comfortably**, starting from the relaxed eyelids, over the features of your face, into the neck, shoulders, arms, hands, chest, belly, pelvis, legs, right side, left side, all the way down to the feet, at your own pace.
The idea **isn't necessarily to follow my pace**, the idea is simply to be able to let the relaxation settle in comfortably at your own pace. There you go, very good. **Doing nothing.**

Let your **breathing** gently accompany you… into this **deep relaxation**
Where your whole body … becomes **completely and totally relaxed** … completely and totally relaxed, …. your body and mind relax… loosen…
free of all tension, all stress, all restraint…

And you give yourself these few **moments of rest**…. in complete safety
pleasantly enjoying this feeling of quiet and calm ….. in fact, having no pressure of any kind … **no obligation**… no one to satisfy … no one to please…

[The body becomes heavy]

Perhaps as you relax, as you unwind, you can let **this body become heavier and heavier**, as if it were finally **sinking** pleasantly into the chair, into the bed.

You can simply let this body sink comfortably into the bed, a bit **like when you fall asleep at night,** a bit like when the body **lets go** and settles comfortably into the bed to begin recovering from the day.

The idea is that as you breathe in the way you are doing, **observing** your body relaxing, you let this body deepen its relaxation. And your mind **has nothing to do,** it just has to observe the body letting go, in the same way as at night when you **fall asleep**.

And your **body knows** that this **rocking** movement you bring to it with your breathing is **comfortable** and pleasant for it. Unconsciously, the body knows that this rocking is something **that brings relaxation**, like when you were in your mother's womb, like when you were rocked on a swing, like when your parents took you in their arms to rock you, to soothe you, to relax you, to calm you down.

Your body ultimately knows what's good for it. Simply let this **body sink comfortably into this state of relaxation**, into this state of relaxation, into this pleasant hypnosis.
From now on, I invite you **to do nothing**. Don't even try to relax, let the body do it, don't force the relaxation, you simply have to let it happen. **Save the energy you might use to deepen the relaxation** for later.

Your body knows what's good for it, you simply let it settle pleasantly into this state of relaxation without doing anything, just observing what's happening, being there, present. **There you go, very good.**

Deepening
From now on, with every **exhale**, this will be a **signal** for your body to settle more comfortably, more deeply into this state of relaxation.
From now on, with every **exhale**, this will be a signal to **double the relaxation** of this hypnosis.

With every exhale, you will go 2 times deeper into this state of hypnosis.
With every exhale, you will go 2 times deeper into this state of hypnosis.

[Body-mind dissociation]
You can **let the hypnosis do its work** and simply observe this relaxation quietly deepening without doing anything.

While your body relaxes more and more and does what it needs to recharge, you can now **take care of your mind.**

We're now going to let this mind relax pleasantly. To do that, I invite you to let an **image of a pleasant place** come to mind, comfortable, any place at all. Let your **unconscious** decide on a place to visit.

Perhaps a place from your **past**, perhaps a place in your **present** or your **future**, perhaps a memory of a **vacation**, perhaps a future trip you'd like to take, I don't know, it's up to you, any place at all.

Simply let your mind offer you a comfortable and pleasant place, right now, without doing anything.

Once your **unconscious** has selected this place, this image, this experience, it can let us know…

Either by having you take a big **breath**,
Or by raising the index finger of your right or left hand
To signal to both of us

That it has had time to select … from the vast reservoir of your memories … a truly pleasant… enjoyable… moment…
That you'll be able to revisit with pleasure …
(wait for the response…) Good, thank you…

As soon as you have this image in mind, this place, I invite you to **observe with your inner eyes** the place you're in. To **welcome** the environment you're in, as in a dream.
Look to the right, look to the left, look ahead, look behind, look up, and the idea here is not to force yourself to look at what's happening, the idea is to let the images penetrate your retina.

[VAKOG]
The idea is to let the **images penetrate your retina**, **do nothing**, don't make any effort to look at what there is to see, simply let the image come and penetrate your retina, to bring an image of the place to your brain, don't force it, don't do anything, you can look at the place you're in through your inner eyes.
First noting whether you're outside or inside.

Whether it's day or evening… or night
Whether you're alone or with others
And little by little, you can note the **environment** you're in…
All the details around you…
And I don't know in what order…

Perhaps note the **sound environment**…
The noises… the sounds … the silence…
Let the sounds come and penetrate your **eardrum**, your inner ear, to stimulate the auditory receptors, do nothing, simply let your inner ears listen to the sounds coming from the right, from the left, from in front, from behind, from above.
And the more you hear the sounds, **the more sensitive you become** to the sounds of the place, **the less you are in this** room, my voice becomes more distant. You can let part of you follow my voice, and let the other part focus on your experience, on the sounds surrounding you in your place.

A bit like being in a **crowd of people**, talking with one particular person, you're attentive to the conversation, but you still hear the background noise around you, everything around you, without having to pay attention to it.

Let my voice be that background noise, and simply pay attention to the pleasant sounds, to your pleasant place.

You may perhaps also feel the **temperature** where you are. Perhaps there's a **light breeze** lifting the hairs on your arms, tickling you. Perhaps, on the contrary, you can feel the **warmth** of the sun on your skin, on your face. Perhaps you can feel the texture of the clothes you're

wearing, on your skin, on your arms. It’s up to you. It’s your place, let yourself be filled with these sensations.

You can also perceive **smells**…
Perhaps the scents of the place, let your olfactory receptors be stimulated by these smells, and there are the temperatures, perhaps cooler, perhaps warmer, let your skin receptors be sensitized by this temperature.
You might also perhaps perceive **tastes**. Perhaps something you’ve just eaten. Something that lingers gently in your mouth. Perhaps a favorite dish. It’s up to you. It’s your experience

Simply let **your mind welcome all these sensations that make your pleasant place what it is.**

[3-minute walk]

If **you’re comfortable where you are**, you can also stay there. It’s up to you. If that’s the case, you’ll hear less of my voice for the next few minutes, so you can focus on your experience and sensations.

Otherwise, I suggest you **take the grand tour** of this place, that is, visit the place your unconscious has chosen for you.

Personally, I particularly enjoy wandering and **getting lost in the villages** I visit, to get to know them better, that village, you see, a bit like you can also do in places like museums.

The idea isn’t **necessarily to know where you’re going**, it’s simply to enjoy getting lost in every nook and cranny of the place to discover it better, to grasp it better.
I simply invite you to **get lost in your place in a pleasant way**, meaning not trying to get somewhere, but to wander, to stroll, to enjoy yourself, to move around the place to discover it better.
You might perhaps **better sharpen each of your senses** to perhaps see other possibilities in the place, to hear certain different sounds, perhaps discover certain different smells, perhaps even different temperatures.

And I invite you here, over **the next three minutes** that will pass, to wander around this place, to stroll, simply to settle in better. And with every second that passes during these minutes, you can simply allow yourself to go **deeper into this state of hypnosis.**
Let your mind settle more deeply into this state of hypnosis with every step you take in the place. And over the next three minutes, you will settle more deeply into this place of relaxation.

On one hand, the **body continues to deepen its relaxation**, its relaxation with every exhale, and on the other hand, you will go **deeper into this state of hypnosis**, on the level of the **mind**, on the psychological level, with every step you take in the place.

Deepening #2
As you begin your walk, I invite you to **count down your first steps**, the first ten steps. With every count, you can go even deeper into this state of relaxation, into this state of hypnosis, and be even more **present** in the place you’re in, in your mind, in your **imagination**.

10, 9 — Deeper.

8 — Deeper.
7 — Deeper.
6, 5 — Twice as deep.
4 — Four times as deep.
3, 2, 1 — Even deeper, and 0.

You can go as deeply into this state of hypnosis as your mind allows. As far as you want. **Everyone has their own level of hypnosis, their own depth.**
You can experience it. Keep wandering, keep strolling.

And for the **2 remaining minutes of this walk** you're taking in the place, **I'll stay quiet** so you can perhaps settle in even better, grasp the experience of your imagination even better. I'll stay quiet for 2 minutes while you stroll quietly, comfortably in the place of hypnosis.

2 minutes of silence

Simply let your mind discover the place you're in, to settle in more **deeply, more calmly, more pleasantly.**

[Choosing a place to meditate]

And perhaps, for a while now, as you've been wandering, strolling in this place, perhaps you've had the chance to discover a much more **specific place**, perhaps a place of safety, a place of **meditation**.
It's like **Buddhists** who sometimes go up into the mountains to meditate in silence, calmly. It's like people who like to **settle by the water** to stop for two minutes and observe the scenery.

The idea of this meditation spot that you may have already found while wandering is that it's like one of **the most beautiful places to see** where you are.

One of the places that feels good **to settle into**. Perhaps by the **water**, perhaps on a **hill**, perhaps on a **bench**, sitting at the foot, **at the top of the mountain**, on a **couch**, in an **armchair** at home, in a room, it's up to you, simply to take a few minutes to meditate quietly, comfortably.

You can try to settle in without forcing it**, without doing anything**, in the place where you can feel safe.

This is the moment when our mind can simply **let go**, can **relax**.
The idea of a meditation place is exactly that: being able to settle into a place similar to this one where your mind **can allow itself not to be alert,** to rest, to do nothing. You can try to find this place; perhaps, during your visit to a pleasant place so far, you've already noticed it and can settle in there.
Settle in.

[Meditation]

The experience of meditation is ultimately the experience of **perfect depth**, this relaxation, this ease. While you meditate in your pleasant place, this is the moment when you can finally simply **feel the deepest relaxation of the experience.**

From the moment you feel safe, you can let your mind surrender to go as deeply as possible into your state of hypnosis, and your brain allows this to the extent that **you are safe.**

You **no longer need to be alert**, you **no longer need to control** anything, whether at the level of your body or your thoughts, **nothing more is needed.**

The idea is to be settled there, in this meditation place, and **to truly do nothing at all.**
During the minutes of meditation that you're going to spend there, you can, on one hand, test your ability to **go as deep as possible into** your state of consciousness, entering as deep a state of hypnosis as your mind allows, and on the other hand, you can simply, during these few minutes, **let your mind forget what needs to be forgotten.**

[Erasing tensions]

Help your mind do nothing, **erase what isn't useful**. While you do this, I'm simply going to talk to you about tensions.
The idea of the experience is that, ultimately, we always have **tensions**. To be able to simply experience relaxation, you need to have experienced tension. **If I clench my fist** and then release it, my hand is relaxed because I felt the tension. And that moment of intense relaxation you feel when you release a tension is something that brings a great deal of comfort. And once you're in the comfort of relaxation, you've completely forgotten the tension. **Tension is the past**, it's something that is **forgotten**.

Right now, it's as if your body has just let go, as if your body were **like my fist.**

It has just released and can now allow itself **to forget this whole part of tension that preceded it.**

While you're having this experience, it's as if you were simply doing a **restoration**, **erasing the tensions** you experienced before **the exercise**. Today, this morning, yesterday, last week, last month, previous years.

While you meditate, you can simply **erase from your mind**, forget these tensions that have **become useless**.

Forget the experiences of tension you had in the past. Simply remember the relaxation. When relaxation sets in, you can forget the rest.
I invite you, during your meditation, to forget what needs to be **forgotten**, as if, finally, the **negative events of the past could be erased**.

Hypnotic Suggestion

[Rub the thumb and index finger]

And while you're restoring your mind, forgetting what needs to be forgotten, remembering the benefits more, I simply want to **invite you to rub the thumb and index finger** of one of your hands, perhaps the right or the left, it's up to you.
And while you make this gesture, rubbing the thumb and index finger of this hand, this is ultimately a **signal** for your **unconscious**, **to be able to record**, to anchor this deep and pleasant

relaxation you are experiencing right now. It's like a **recorder**. Rub the thumb and index finger of this hand, right or left, it's up to you.

It's a signal for your unconscious, **to record the experience of relaxation,** of ease, to anchor it in your unconscious.

The **next time you make this gesture**, rubbing the thumb and index finger of this hand, it will be a signal for your unconscious to reactivate **more quickly and more deeply** this state of safety, of meditation, of comfort.

The next time you rub the thumb and index finger of this hand, it will be a signal for your unconscious to **reactivate more deeply** this feeling of **safety**, of **meditation**, of **comfort**, as deeply as your mind allows.
You may perhaps go **even more deeply** into this state of relaxation the **next time**, because the experience is as if your **unconscious has shown you the way** to access this experience of comfort, safety, and relaxation. There you go, very good.

And you can perhaps continue to enjoy **several more minutes** of this meditation, doing nothing, simply letting memories fade, **those that aren't useful**, ultimately replacing these useless memories with **new positive memories**, perhaps relaxation strategies, perhaps rest, perhaps dreams, perhaps beautiful images, I don't know, it's up to you.

I'm now going to stay quiet for **the next two minutes**, and during these next two minutes, two things will happen.

1. The first thing is that you will continue to **meditate** quietly and pleasantly in your imaginary place.
2. The second thing is that while you meditate, **your unconscious will store** all the benefits of the exercise you did today, the relaxation, the depth of the relaxation, the safety, the comfort, and the signal, the switch, that you **can use at any time.**

I'm going to stay quiet for the next two minutes.

2 minutes of silence

You can take your meditation time to be comfortably settled in the place, to mobilize your unconscious resources, to facilitate forgetting, to do nothing.

And gradually, perhaps you can allow yourself to quietly come out of your meditation, to now **stand up in your hypnosis and continue strolling in the pleasant place.**

It's as if, finally, after being able to watch the sunset by the sea, a prestigious painting in a museum, you now allow yourself to leave the museum room, to leave the shore, **to set off again**, perhaps to discover other places on the beach, perhaps to discover other rooms in the museum, I don't know, it's up to you.

Ultimately, the idea is that this **meditation** place you found in your hypnosis is a place you can return to at any time. It's your unconscious that led you into this part of your mind, into this meditation place. **You can trust your unconscious.**

Return to the waking state

And as you start moving again, perhaps you can simply prepare to **return to the waking state** in a few moments.

And for that, I'm simply going to **accompany you through it**. Like before, I'll count down, this time **from 0 to 10,** so you can climb back up through the different levels of your state of consciousness and find a waking state, to comfortably and pleasantly resume the course of your day. As you walk comfortably in the place, we're going to simply begin the countdown.

1. You can now simply feel your **breathing** becoming more and more normal as time passes.
2. The **heart** returns to a normal rhythm.
3. Perhaps you can better feel the **position** of your body in the chair or in the bed. Feel every corner of the bed, the contact with your body. Your **awareness** of the room quietly returns.
4. Perhaps you can now smell other **scents** in the room and distinguish them from the smells of the place you were in.
5. Perhaps you can now better hear my **voice** or the **sounds** of the room you're in.
6. Perhaps you can now feel a bit of **muscular** movement, of tone, perhaps feel movements in your body.
7. Perhaps now, you feel your **breathing** is almost normal. You feel even more **the weight of this body** becoming light again, more present, at least, in your mind.
8. **Breathing** becomes normal. Through your closed eyelids, you see the light of the room.
9. You can now let three big breaths in come. And with each breath, you'll climb back up even more quickly through the different levels of this hypnosis to quietly, comfortably find the room you were in and resume the course of your day.

Everyone at their own pace. Let very big breaths come now.
There you go.

And gradually come back into the room you were in so we can quietly resume the course of the day.

And you wake up **as if, finally, you were waking up on a Sunday morning** when there's nothing to do, when perhaps you smell the good scent of croissants in the kitchen with the coffee. There you go. You can simply **come back at your own pace.**

File SM3. **Thematic Tree — Code List — Qualitative Analysis, HYlaMI Project**

Inter-rater Revision

1. **KNOW_IMPRESS_HYP :** Knowledge and impressions regarding hypnosis
    - **1.1. HYP_KNOW_PRIOR_EXP** — Prior knowledge and experiences with hypnosis
    - **1.2. HYP_IMPRESS_AFTER_PRESENT** — Impressions / attitude toward hypnosis after the program presentation
    - **1.3. HYP_IMPRESS_EXERCISE** — Experience during the hypnosis exercise and views on medical hypnosis after the exercise
        - **HYP_VIEW_AFTER_EXERCISE** — Views after completing the exercise
        - **HYP_CHANGE_IN_VIEW** — Change in views on hypnosis following the experience
        - **HYP_SENSATIONS_EXERCISE** — Sensations experienced during and after the hypnosis exercise
        - **HYP_DIFFICULTIES_ENCOUNTERED** — Difficulties encountered during the hypnosis exercise
        - **HYP_ADHERENCE** — Adherence to the method following the hypnosis exercise
2. **REL_ACC_PROG :** Relevance and acceptability of the program (migraine management) — How well the program seems suited to people with migraines
    - **PROG_IMPRESS** — Impressions of the program after the presentation and hypnosis exercise
    - **REL_NB_SESSIONS** — Number of sessions
    - **REL_SESSION_DURATION** — Duration of sessions
    - **REL_THERAPEUTIC_TARGETS** — Therapeutic targets of the program
    - **REL_TIMING** — Relevance of the timing of hypnosis or intervention delivery
        - **REL_TIME_OF_DAY** — Time of day for delivering the intervention
        - **REL_HYPNOSIS_TIMING** — Best timing for administering hypnosis
    - **REL_EXERCISE_CHOICE** — Choice of hypnosis exercises
        - **REL_EXERCISE_CONTENT**
    - **REL_NB_PARTICIPANTS** — Number of participants (adequacy)
    - **REL_TELEPRACTICE** — Telepractice delivery modality
    - **REL_IDEAL_CLINICIAN** — Characteristics of the ideal clinician
    - **REL_IMPROVEMENT_SUGGESTIONS** — Suggestions for program improvement
    - **REL_OTHER_COMMENTS** — Other comments on the relevance and acceptability of the program
3. **PROG_EFFECTS :** Expected and perceived effects of the program
    - **EFF_STRESS_ANXIETY_DEPRESSION** — Expected or perceived effects on anxiety-depressive symptoms and stress
    - **EFF_RELAXATION** — Expected or perceived effects on relaxation

EFF_PAIN — Expected or perceived effects on pain, its management, or perception

EFF_MEDICATION — Expected or perceived effects on medication use

EFF_MIGRAINE_FREQ_INT_DUR — Expected or perceived effects on migraine frequency, intensity, or duration

EFF_DAILY_FUNCTIONING — Expected or perceived effects on daily functioning

EFF_OTHER_EFFECTS — Other expected or perceived effects

4. **EMERGENT_SURPRISE :** Unanticipated elements not captured by the initial thematic tree (surprises, findings, emerging elements)

Table SM 4. Medications reported by participants at the time of the study (N = 17).

Therapeutic class	Medications reported
Acute treatment — analgesics and NSAIDs	Acetaminophen (Tylenol); ibuprofen (Advil); naproxen (Aleve, Naprosyn); diclofenac (Voltaren, Cambia); butalbital–acetylsalicylic acid–caffeine (Fiorinal); corticosteroid, molecule unspecified
Acute migraine treatment — triptans	Zolmitriptan (Zomig, oral and nasal); sumatriptan; rizatriptan; frovatriptan; naratriptan; almotriptan
Acute migraine treatment — gepants	Ubrogepant (Ubrelvy)
Migraine prophylaxis — anti-CGRP monoclonal antibodies	Galcanezumab (Emgality); fremanezumab (Ajovy); eptinezumab (Vyepti)
Migraine prophylaxis — other agents and procedures	Nadolol; candesartan; onabotulinumtoxinA (Botox); local injections (infiltrations); leuprolide depot (Lupron)
Antidepressants and other psychotropics	Amitriptyline (Elavil); nortriptyline (Aventyl); venlafaxine and venlafaxine XR (Effexor); desvenlafaxine; citalopram; sertraline (Zoloft); trazodone; quetiapine
Neuromodulators, muscle relaxants and hypnotics	Pregabalin (Lyrica); cyclobenzaprine; nabilone; zopiclone
Hormonal treatments	Estradiol; medroxyprogesterone; norethindrone (Jencycla)
Cardiometabolic	Metformin ER; atorvastatin (Lipitor); lipid-lowering agent, molecule unspecified (Sandoz)
Gastrointestinal	Dexlansoprazole (Dexilant); pantoprazole; ranitidine
Respiratory	Salbutamol (Ventolin); fluticasone (Flovent)
Antihistamines and antivertigo agents	Antihistamine, molecule unspecified; betahistine; dimenhydrinate (Gravol)
Supplements and natural health products	Riboflavin (vitamin B2); magnesium; coenzyme Q10

Note. Medications were self-reported by participants in the pre-visit clinical questionnaire and were not verified against medical records or pharmacy records.

Table SM 5. Verbatims for Themes and Sub-themes

Themes and Sub-Themes	Selected Verbatims
**Theme 1. Knowledge and impressions regarding hypnosis**	
1.1. Prior knowledge and experience with hypnosis (17/17)	• P17: "I don't really know much about hypnosis apart from what you see on TV." P14: "I tried to do some research, but […] I mostly found people who had […] a […] less scientific approach […]. I felt less comfortable; I wanted something with more solid grounding." P3: "I completed an introductory training in hypnosis as part of my work as an occupational therapist."
1.2. Impressions / attitude toward hypnosis after the program presentation (13/17)	• P14: "The fact that you showed little images [of brain scans], that there have been official scientific studies, in my case, that helps me trust the method." P16: "It's not negative, it's not positive, in the sense that I've never tried it."
1.3. Experience / views on hypnosis during / after the hypnosis exercise	
1.3.1. Views on hypnosis after the exercise (12/17)	• P7: "It's non-invasive, it's only positive, so it's worth trying."
1.3.2. Change in views on hypnosis following the experience (14/17)	• P14: "I had no idea it was a capacity we naturally have. That's good too. I think it opens the door to making it a viable option."
1.3.3. Sensations experienced during / after the exercise (16/17)	• P12: "But I really am more relaxed. It's as if I had slept for several hours. I feel truly rested." P17: "I never reach a state like the one I reached there [when I meditate], that was wonderful. I'd do it again." P3: "You can't really multitask, [so] you're forced to focus on your voice more than on your problems." P8: "It really allowed me to find a little corner of memory, a paradise, that does me good, that is soothing."
1.3.4. Adherence to the method following the exercise (6/17)	• P6: "But I'm certainly interested in continuing. Not only for migraine, but for anxiety too. I think the two are interconnected."
**Theme 2. Relevance and acceptability of the program**	
2.1. Impressions of the program (17/17)	• P14: "I think it's really something we need. I found this project extraordinary." P8: "It brings a tool for managing our emotions to truly learn to prevent or treat [pain] during the pain itself."
2.2. Number of sessions (14/17)	• P16: "8 hours somewhere in a lifetime is almost nothing. […] I think the number is perfect, and it will perhaps give more possibilities to choose the one that works best." P17: "If there were twice as many, it would make me just as happy."
2.3. Duration of sessions (13/17)	• P5: "I used to have one-hour sessions with a psychologist, so I think it's well-adapted. It's not too long."
2.4. Therapeutic targets of the program (13/17)	• P7: "Whether it's through hypnosis, whether it's about making the person more functional, whether it's through small daily habits, that might seem trivial to someone who is healthy, but for someone who is very ill, […] it's important, it's precious." P5: "When you have a migraine, you become so discouraged that you end up crying for no reason."
2.5. Relevance of the timing of hypnosis or intervention delivery	
2.5.1. Best time of day for delivering the intervention (15/17)	• P12: "For me, I think the morning is still better because in the evening, you're more tired."
2.5.2. Best timing for administering hypnosis (5/17)	• P6: "That's a good question. I'd say all three. At the beginning, when you feel a migraine starting, you anticipate a lot of things. It might help you calm down a little. When you're at the peak of the pain, it can help you focus on something else. And when you're in the post-migraine phase, I think it could be important too, because when you're in that last phase, the postdrome, you can really be more prone to starting another episode. So maybe, to preserve that state of calm and well-being, to prevent another attack from starting."

2.6. Choice of hypnosis exercises (16/17)	• P3: "It [would be good] if there were a session we could do post-migraine, a reactivation one […] to truly regain control over my body." "There's something else that also really bothers me during a migraine, it's my sensitivity. Even physical sensitivity; being touched is complicated for me. I don't know if we could have a session focused on that, to lower our defences […] make us feel less physically irritable." P10: "Learning to pace yourself, even on a good day, learning not to go overboard."
2.6.1. Relevance of exercise content (8/17)	• P14: "It could be an environment like a forest, or something that engages all the senses [like water], so that it feels as though you're melting into something that is not you, as if there were no boundaries between your body and the surrounding environment." P4: "I had the little hamster running around from time to time. I'd find a place, and then whoops, I'd jump to another. […] I didn't have any trouble forgetting my body enough to relax properly, but the thoughts, that's the problem." P14: "If I don't have some mechanism that wakes me up when the pain starts so I can take my medication and stop the attack, if I don't wake up, by the time I get up in the morning it's often too late."
2.7. Number of participants (15/17)	• P12: "Since it's on Zoom, […] the number of participants shouldn't really be felt, except at the end of the session."
2.8. Telepractice delivery modality (17/17)	• P1: "You can have someone with the same condition as me but who lives in Abitibi, and we can all be together."
2.9. The ideal clinician (17/17)	• P10: "For me, it really comes down to just the voice." P15: "I would picture a woman, because it is much more a disease that affects women."
2.10. Suggestions for improvement (5/17)	• P14: "If people want to learn more about hypnosis […] if there are books people could read to find out more, if there's a documentary, articles on the topic, or maybe links to studies that have been done, because that can support […] [program] adherence."
2.11. Other comments (7/17)	• P3: "I like the fact that the hypnosis is also given back at the end, each time, so that the person can continue doing it on their own, at home. I think that's great."
**Theme 3. Expected and perceived effects of the program**	
3.1. On anxiety-depressive symptoms and stress (16/17)	• P12: "If we can remove the stress that migraine creates when you're having one, it would certainly lift a heavy burden from the migraine itself."
3.2. On relaxation (12/17)	• P4: "After a migraine, it's as if a train had run over you, the muscle pain and everything. So these are good exercises to help manage that."
3.3. On pain, its management, or perception (7/17)	• P11: "But in the moment, you definitely feel a relaxation, a reduction in pain." P10: "It will mean we'll be able to manage [the pain]."
3.4. On medication use (14/17)	• P13: "On days when I have a headache but don't want to take medication so as not to take too much, well, taking the time to do this would do me good."
3.5. On migraine frequency, intensity, or duration (13/17)	• P9: "Above all, an improvement in pain management. Perhaps an improvement in how often I need to take medication, if the migraines are less frequent, you take less medication. And when you do take it, it's more effective."
3.6. On daily functioning (16/17)	• P7: "I think it can improve quality of life."
3.7. Other expected or perceived effects (2/17)	• P14: "We [could] give our body that sense of security it needs. And that can make us more resilient in our daily activities."
**Theme 4. Unanticipated elements** (9/17)	• P15: "It's almost as bad as the pain itself, that kind of incomprehension people have about it." "I would suggest, at any rate, that the two worlds talk to each other more, so that it becomes more a part of what the neurologist, the migraine specialist treating us, has to offer." P12: "I'm certain that most people living with this would be glad to know there might be a more natural alternative."
